# Terroir Rediscovered: A Polyphasic Approach to Autochthonous Lactic Acid Bacteria as Functional and Microbial Heritage Resources in Portuguese PDO Cheeses

**DOI:** 10.3390/foods15183322

**Published:** 2026-09-19

**Authors:** Susana Serrano, Joana Monteiro Marques, Cinthia Alves-Barroco, Susana Morais, Catarina Araújo, Jéssica Elias, Maria Teresa Barreto-Crespo, Teresa Semedo-Lemsaddek

**Affiliations:** 1 CIISA—Center for Interdisciplinary Research in Animal Health, Faculty of Veterinary Medicine, University of Lisbon, 1300-477 Lisbon, Portugal; sserrano@fmv.ulisboa.pt (S.S.); jmarques@fmv.ulisboa.pt (J.M.M.); claraujo@fmv.ulisboa.pt (C.A.);; 2 AL4AnimalS—Associate Laboratory for Animal and Veterinary Sciences, 5000-801 Vila Real, Portugal; 3 INIAV—National Institute of Agricultural and Veterinary Research, I.P., 2780-157 Oeiras, Portugal; 4 UCIBIO—Applied Molecular Biosciences Unit, Department of Life Sciences, NOVA School of Science and Technology, Universidade NOVA de Lisboa, 2829-516 Caparica, Portugal; caabarroco@gmail.com; 5 Associate Laboratory i4HB, NOVA School of Science and Technology, Universidade NOVA de Lisboa, 2829-516 Caparica, Portugal; 6 Laboratory of Molecular Microbiology of Human Pathogens, ITQB NOVA—Instituto de Tecnologia Química e Biológica António Xavier, Universidade Nova de Lisboa, 2780-157 Oeiras, Portugal; susana.morais@itqb.unl.pt; 7 iBET—Instituto de Biologia Experimental e Tecnológica, Av. República, Qta. do Marquês, 2780-157 Oeiras, Portugal; tcrespo@ibet.pt; 8 ITQB—Institute of Chemical and Biological Technology António Xavier, Nova University of Lisbon, Av. República, 2780-157 Oeiras, Portugal; 9 BioISI—Biosystems & Integrative Sciences Institute, Faculty of Sciences, University of Lisbon, 1749-016 Lisbon, Portugal

**Keywords:** Portuguese traditional cheeses, autochthonous lactic acid bacteria, microbial heritage, polyphasic characterization, technological properties, probiotic potential, food safety, starter and adjunct cultures

## Abstract

Portuguese Protected Designation of Origin (PDO) cheeses from Azeitão and Nisa harbor unique autochthonous microbial communities integral to their terroir, sensory identity, and authenticity. Rediscovering and preserving this microbial heritage is essential for safeguarding traditional cheesemaking practices and unlocking the biotechnological potential of indigenous microorganisms. This study used a polyphasic approach to isolate, preserve, and functionally characterize lactic acid bacteria (LAB) from these cheeses, aiming both to conserve autochthonous microbial resources and evaluate their suitability for food applications and preliminary probiotic-related traits. From 2304 bacterial isolates, 119 representative LAB were selected and screened for safety based on hemolytic and gelatinolytic activities and antimicrobial susceptibility. Technological potential was assessed through acidification, proteolytic and lipolytic activities, and antagonism against foodborne pathogens, while probiotic traits were evaluated via tolerance to acidic pH and bile salts, survival under simulated gastrointestinal conditions, and auto- and co-aggregation. Thirty-nine isolates exhibited desirable technological traits, including proteolytic and lipolytic activities and broad-spectrum pathogen antagonism. Among these, three isolates (two *Leuconostoc mesenteroides* and one *Enterococcus hirae*) combined desirable technological traits with survival under simulated gastrointestinal conditions, representing preliminary multifunctional candidates. These findings reveal *Azeitão* and *Nisa* PDO cheeses as living reservoirs of terroir, where indigenous LAB rediscovered through this approach represent both a key component of Portugal’s microbial heritage and a source of functional cultures for sustainable dairy innovation.

## 1. Introduction

Traditional cheeses represent more than mere food products; they are deeply rooted in their territory of origin, symbolizing the history, culture, and ancestral knowledge of producing communities. In Mediterranean countries like France, Italy, Spain, and Portugal, these production systems rely on biological processes that directly influence the overall quality and safety of the final product [1]. To protect and preserve this cultural and economic heritage, the European Union established geographical indication schemes, such as Protected Designation of Origin (PDO) and Protected Geographical Indications (PGI) [2]. Currently, 11 regions in Portugal produce cheeses according to PDO specifications, meaning that the raw ingredients come from the region of origin where all steps of production take place. Scientific efforts to decode the microbial consortia and ecological dynamics of these traditional products have focused heavily on conventional microbiology. Among the most extensively characterized Portuguese cheeses are *Serra da Estrela* cheese, studied for its baseline microbial communities and their evolution during cheese maturation, and *Beira-Baixa* (*Castelo Branco* and *Picante*), *Pico*, *Serpa* and *São Jorge* cheeses. In contrast, significantly less is known about *Rabaçal*, *Terrincho*, *Évora*, *Azeitão* and *Nisa* cheeses. Regarding the latter two, the existing literature consists largely of master’s theses or articles focused on specific bacterial groups rather than complete ecological profiles, with the exception being the metataxonomic-based approach performed by Serrano et al. (2026) [3]. Both *Azeitão* and *Nisa* cheeses are produced using artisanal protocols, raw ewe’s milk (*Merina Branca* breed in *Nisa*), plant-based coagulant *Cynara cardunculus* L. and salt [4,5]. The use of raw milk, combined with traditional “know-how” and producer-specific microbiota, creates a complex microbial ecology that defines the distinctive organoleptic features appreciated by consumers [6]. Because no commercial starter cultures are added during manufacture, the characterization and preservation of this autochthonous microbiota are essential for maintaining the production and authenticity of these products.

Lactic acid bacteria (LAB) have great influence on the quality and safety of Portuguese PDO cheeses, offering multifunctional benefits to the final product. This group encompasses a diverse community of both starter and non-starter LAB, including genera such as *Lactococcus*, *Leuconostoc*, members of the *Lactobacillaceae* family (*Lactiplantibacillus*, *Lacticaseibacillus*, *Limolactobacillus* or *Levilactobacillus*), and *Enterococcus*. These microorganisms are active throughout all production stages: starter LAB (SLAB) (*Lactococcus* and some species of the *Lactobacillaceae* family) initiate milk acidification, while non-starter LAB (NSLAB) (*Lactobacillus*, *Leuconost* and *Enterococcus*) contribute to the development of aroma and flavor through complex proteolytic and lipolytic activities [7]. Beyond its sensory contributions, LAB contributes to food safety by producing antimicrobial metabolites like organic acids and bacteriocins. These compounds inhibit the growth of pathogens such as *Listeria* and *Staphylococcus*, which are critical concerns in raw dairy products [8].

Raw milk products are rich sources of LAB with a multitude of functional properties. However, because these products retain the autochthonous microbiota inherent to the milk, a rigorous safety assessment is mandatory, as contamination by opportunistic pathogens can occur during milk harvest or storage [1]. The European Food Safety Authority (EFSA) serves as the primary agency for scientific risk assessment in the European Union, providing independent advice to inform food safety regulations and the authorization of microorganisms used in the food chain [9]. To organize the selection of microorganisms for industrial use, EFSA developed the Qualified Presumption of Safety (QPS) concept. This framework allows for a safety pre-evaluation at the taxonomic unit level based on four main pillars: taxonomic identification, the body of knowledge, pathogenic potential, and intended use [9]. The QPS list is updated triennially and represents an assessment framework that acknowledges the history of safe use while ensuring the absence of safety concerns, such as acquired antimicrobial resistance (AMR). Several LAB genera and species hold QPS status and are widely utilized in the food industry, including members of the *Lactobacillaceae* family (former *Lactobacillus* spp.), *Lactococcus lactis*, *Leuconostoc* spp., and *Streptococcus thermophilus*. In contrast, the genus *Enterococcus* spp., though prevalent in traditional cheeses and a significant contributor to ripening, is excluded from the QPS list [10]. Consequentially, these bacteria require an extensive case-by-case safety assessment regarding virulence factors and antibiotic resistance, to ensure consumer safety. Furthermore, while the technological benefits of these isolates are well-documented, there is a growing interest in their functional attributes. Identifying strains that not only excel technologically but also possess probiotic potential, such as the ability to survive conditions in the gastrointestinal tract, represents a significant opportunity to develop health-promoting functional foods.

Despite increasing knowledge of the microbial dynamics occurring during the ripening of Portuguese PDO cheeses, the autochthonous microbiota of *Azeitão* and *Nisa* PDO cheeses remains insufficiently explored. Beyond taxonomic characterization, preserving these indigenous microbial communities is essential to safeguard the microbial heritage that underpins the authenticity, typicity, and terroir of these traditional products. At the same time, systematic characterization of these microorganisms may reveal strains with valuable technological and probiotic traits, enabling their future application while maintaining the link with their geographical origin.

Therefore, the primary aim of this study was to isolate, preserve, and establish a representative culture collection of the autochthonous microbiota associated with Azeitão and Nisa PDO cheeses, as a strategy for rediscovering and conserving their microbial heritage. Subsequently, a polyphasic characterization was performed to identify lactic acid bacteria (LAB) with desirable functional properties. Representative isolates were assessed for safety, technological performance, and probiotic-related traits, including survival under simulated gastrointestinal conditions and auto- and co-aggregation abilities.

By integrating microbial conservation with functional characterization, this work would reveal a hidden dimension of the terroir of Azeitão and Nisa. PDO cheeses, potentially providing both a valuable microbial resource for the preservation of Portuguese cheese biodiversity and a scientific basis for the future development of indigenous functional starter and adjunct cultures.

## 2. Materials and Methods

In this study, LAB isolates were divided into two groups for analytical purposes: *Enterococcus* spp. and non-enterococcal LAB. Although enterococci are taxonomically classified as LAB, they were evaluated separately due to their opportunistic nature and absence of Qualified Presumption of Safety (QPS) status.

### 2.1. Cheese Sampling and Microbial Isolation

Cheese samples were collected from all PDO producers in *Azeitão* and *Nisa*, Portugal, during two production years (2021–2022), comprising four cheese factories in *Azeitão* (A1–A4) and two in *Nisa* (N9–N10). Sampling procedures and microbial isolation were performed as previously described by Bastião Rocha et al. (2022) [11]. All cheese samples were preserved at −80 °C, while isolated bacterial colonies recovered from agar plates were purified by consecutive streaking, resuspended in Buffered Peptone Water (BPW) supplemented with 20% glycerol, and preserved at the same temperature. LAB isolates were isolated and maintained on De Man–Rogosa–Sharpe agar (MRS; Liofilchem, Roseto degli Abruzzi, Italy) and M17 agar (Liofilchem, Roseto degli Abruzzi, Italy). *Enterococcus* spp. were isolated on Slanetz Bartley agar (SBA; Liofilchem, Roseto degli Abruzzi, Italy) and subsequently maintained on Brain Heart Infusion agar (BHI; Liofilchem, Roseto degli Abruzzi, Italy).

### 2.2. Molecular Characterization

#### 2.2.1. DNA Extraction

DNA extraction from pure cultures was performed using the boiling method [12]. Briefly, a colony was suspended in 50 μL of Tris-EDTA buffer supplemented with 0.1% (*v*/*v*) Tween 20 (Merck KGaA, Darmstadt, Germany) and incubated, at 100 °C for 10 min. Immediately after, the suspensions were placed on ice for 10 min, inducing thermic shock, followed by a centrifugation at 14,000 rpm for 5 min in an Eppendorf^®^ 5415R centrifuge (Eppendorf, Hamburg, Germany). The supernatants were used directly in PCR reactions and stored at −20 °C.

#### 2.2.2. Genomic Diversity—RAPD PCR

Screening for representative isolates was carried out by RAPD-PCR using the primers OPC15 (5′-GACGGATCAG-3′) and (GTG)_5_ (5′-GTGGTGGTGGTGGTG-3′) in independent reactions [13]. For each primer, a reaction mixture with a total volume of 20 μL was prepared as follows: 10 μL of NZYTaq II 2x Green Master Mix (NZYTech, Lisboa, Portugal), 7 μL of Milli-Q water (Millipore, Merck KGaA, Darmstadt, Germany), 2 μL of DNA and 1.0 μL of primer at 50 pmol/μL (STAB VIDA, Caparica, Portugal). Amplification was performed on a thermocycler GTC96S (Cleaver Scientific, Rugby, UK), under the following conditions: 94 °C for 5 min, followed by 40 cycles consisting of 94 °C for 1 min, annealing at 40 °C for 2 min, extension at 72 °C for 2 min, and a final extension at 72 °C for 10 min. Genetic profiles were resolved by electrophoresis on 1.2% agarose gel (GeneOn, Groß-Rohrheim, Germany) using 0.5× TBE buffer (4.5 × 10^−2^ M Tris, 4.5 × 10^−2^ M boric acid, and 1 × 10^−3^ M Na_2_EDTA, NZYTech, Lisboa, Portugal). Wells were loaded with 8 μL of amplification mixture, 2 μL of GelRed 10× (Biotium, Fremont, CA, USA), and 2 μL of bromophenol blue 6× (Merck KGaA, Darmstadt, Germany). Electrophoresis was performed at 90 V for 3 h. Gel results were observed in ChemiDoc XRS+ device (Bio-Rad, Hercules, CA, USA) with Image Lab 6.1. software (Bio-Rad, Hercules, CA, USA). Reproducibility was assessed by testing 10% replicates, selected at random.

#### 2.2.3. Data Analysis

RAPD profiles were analyzed using the BioNumerics software (version 6.6.5, BioMérieux, Marcy-l’Étoile, France). All gel images were normalized, and dendrograms were generated based on Pearson correlation coefficient using the unweighted pair group method with arithmetic mean (UPGMA). The dendrograms obtained were used to select representative LAB for further characterization. Reproducibility was assessed based on the average similarity among replicate profiles.

#### 2.2.4. Genus and Species Allocation

Genus and species assignment was performed based on the LAB species most frequently reported in Portuguese traditional cheeses [14]. Identification was carried out by PCR amplification using the primers LeucA/LeucS [15] for *Leuconostoc* spp., LbLMA1-rev/R16-1 [16] for *Lactobacillaceae* sp., and Ent1/Ent2 [17] for *Enterococcus* spp., for genus allocation. For species allocation, the following primers were used: His1/His2 [18] for *Lactococcus lactis* subsp. *lactis* and *Lactococcus lactis* subsp. *cremoris*, SCAR-LBR-F/SCAR-LBR-R [19] for *Levilactobacillus brevis*, SCAR-LPL-F/SCAR-LPL-R [19] for *Lactiplantibacillus plantarum*, SCAR-LEU-F/SCAR-LEU-R [19] for *Leuconostoc mesenteroides*, SCAR-FL1/SCAR-FL2 [20] for *Enterococcus faecalis*, FM1/FM2 [20] for *E. faecium*, DU1/DU2 [20] for *E. durans* and Mur1/Mur2 [21] for *E. hirae*. The reaction mixture for each set of primers, with a total volume of 20 μL, was prepared with 10 μL of NZYTaq II 2x Green Master Mix (NZYTech, Lisboa, Portugal); 7 μL of Milli-Q water (Millipore, Merck KGaA, Darmstadt, Germany), 2 μL of DNA and 0.5 μL of forward and reverse primer at 50 pmol/μL (STAB VIDA, Caparica, Portugal).

Reference strains (*Lactiplantibacillus plantarum* DSMZ 20174^T^ (Leibniz-Institut DSMZ—Deutsche Sammlung von Mikroorganismen und Zellkulturen GmbH), *L. mesenteroides* CECT 219T (*Colección Española de Cultivos Tipo*), *Levilactobacillus brevis* DSMZ 20054^T^ *E. faecalis* ATCC 29212 (American Type Culture Collection), *E. faecium* E300, *E. hirae* DSMZ 20160^T^, and *E. durans* DSMZ 20633^T^) were included in all reactions. Additionally, 10% of the isolates (randomly selected) were tested as independent replicates to ensure reproducibility.

Amplification was performed on a thermocycler GTC96S (Cleaver Scientific, Rugby, UK). Different protocols were used depending on the genus/species to be identified. For the *Lactobacillaceae* family and *Leuconostoc* spp., the following conditions were used: 95 °C for 5 min, 35 cycles of 95 °C for 30 s, 55 °C for 30 s, and 72 °C for 1 min, followed by a final extension at 72 °C for 5 min. For *Lactococcus* spp., the following conditions were applied: 95 °C for 5 min, 35 cycles of 95 °C for 30 s, 42 °C for 30 s, and 72 °C for 1 min, followed by a final extension at 72 °C for 5 min.

In the case of *L. mesenteroides*, *Levilactobacillus brevis* and *Lactiplantibacillus plantarum*: 95 °C for 5 min, 35 cycles of 95 °C for 30 s, 55 °C for 30 s, and 72 °C for 1 min, followed by a final extension at 72 °C for 5 min. Finally, for *Enterococcus* spp.: 95 °C for 5 min, 35 cycles of 95 °C for 45 s, 57 °C for 45 s, and 72 °C for 45 s, followed by a final extension at 72 °C for 10 min.

Amplicons were resolved by electrophoresis on 1.2% agarose gels (GeneOn, Groß-Rohrheim, Germany) prepared with 0.5× TBE buffer (4.5 × 10^−2^ M Tris, 4.5 × 10^−2^ M boric acid, and 1 × 10^−3^ M Na_2_EDTA, NZYTech, Lisboa, Portugal). Each well was loaded with 8 μL of PCR product, 2 μL of GelRed™ (10×; Biotium, Fremont, CA, USA), and 2 μL of 6× bromophenol blue (Merck KGaA, Darmstadt, Germany). Electrophoresis was carried out at 90 V for 2 h, and gels were visualized using a ChemiDoc™ XRS+ imaging system with Image Lab 6.1. software (Bio-Rad, Hercules, CA, USA).

Identification of isolates that displayed probiotic potential was confirmed by Sanger sequencing of the 16S rRNA gene regions V1–V9 using the primers 27F (5′-AGAGTTTGATCMTGGCTCAG-3′) and 1492R (5′-GGYTACCTTGTTACGACTT-3′), performed by STAB VIDA (Caparica, Portugal).

### 2.3. Lactic Acid Bacteria Characterization

#### 2.3.1. Safety Evaluation

##### Virulence Traits

Hemolytic and Gelatinolytic Activities

Metabolically active cultures were inoculated onto Columbia agar plates supplemented with 5% horse blood agar (Frilabo, Trofa, Portugal) and incubated under anaerobic conditions using Oxoid Anaerogen (Thermo Fisher Scientific, Waltham, MA, USA) sachets, for 48 h at 37 °C. Hemolytic activity was evaluated and characterized according to Semedo et al. (2003) [22]. Positive (*E. faecalis* MMH594) and negative (*E. faecalis* ATCC 29212) controls were included. Gelatinase activity was tested using gelatine agar plates, prepared as described by Domingos-Lopes et al. (2017) [23]. To check for the presence of gelatinase-positive isolates, after the incubation period (48 h at 30 °C for non-enterococcal LAB, and 24 h at 37 °C for enterococci), a saturated ammonium sulphate solution (4.1 M) was poured onto the plates and incubated for 1 h at room temperature. Isolates surrounded by a transparent halo, indicating gelatine degradation, were considered positive for the tested activity. As control, cheese isolates from *Azeitão* and *Nisa* isolated and characterized in previous years, that were gelatinase positives, were used as positive controls. Additionally, 10% of the isolates (randomly selected) were tested as independent replicates to ensure reproducibility.

Screening for Virulence Determinants

Virulence determinants were evaluated by multiplex-PCR, only for enterococcal isolates. The targeted genes included determinants associated with cell aggregation (*agg*) [24], biofilm (*esp*) [24], gelatinase production (*gelE*) [22], and cytolysin/hemolysin activity (*cylA*) [24]. For PCR, 20 μL reaction mixtures were prepared as follows: 10 μL of NZYTaq II 2x Green Master Mix (NZYTech, Lisboa, Portugal), 2 μL of DNA, 0.5 μL of each forward and reverse primer at 50 pmol (STAB VIDA, Caparica, Portugal), and Milli-Q water (Millipore, Merck KGaA, Darmstadt, Germany). Positive (*E. faecalis* MMH 594) and negative (sterile water) controls were included in all reactions. Thermocycler reaction conditions were as follows: 95 °C for 5 min, 35 cycles of 95 °C for 45 s, 55 °C for 45 s, 72 °C 45 s; and 72 °C for 5 min. To confirm reproducibility, 10% of isolates were randomly selected and re-tested as independent replicates. Subsequently, an aliquot of 8 μL of the PCR products was mixed with 2 μL of GelRed (10×; Biotium, Fremont, CA, USA) and 2 μL of 6× bromophenol blue (Merck KGaA, Darmstadt, Germany) and resolved on a 1.2% agarose gel (GeneOn, Groß-Rohrheim, Germany) by electrophoresis at 90 V for 2 h. Visualization was performed using a ChemiDoc™ XRS+ imaging system with Image Lab 6.1. software (Bio-Rad, Hercules, CA, USA).

Antimicrobial Susceptibility Testing

Antimicrobial susceptibility testing was performed against a total of 14 antimicrobials using the Kirby–Bauer disk diffusion method, with results interpreted according to the microbiological breakpoints defined by Charteris et al. (1998) [25] and Touret et al. (2018) [26] for non-enterococcal LAB, and the Clinical and Laboratory Standards Institute (CLSI) guidelines M100 [27] for enterococci. Briefly, each isolate was suspended in sterile Ringer solution (Oxoid, Hampshire, UK) to a turbidity equivalent to 0.5 McFarland standard (≈10^8^ CFU/mL) and spread-plated onto the appropriate agar medium. Antibiotic disks (Liofilchem, Roseto degli Abruzzi, Italy) were equidistantly placed on the agar surface. Plates were incubated for 18–24 h (enterococci) and/or for 48 h at 37 ± 2 °C (non-enterococcal LAB). Inhibition zone diameters were measured and classified as susceptible or resistant according to the breakpoints applicable to each bacterial group.

For non-enterococcal LAB, the following antimicrobials were tested: vancomycin (30 µg), ampicillin (10 µg), tetracycline (30 µg), kanamycin (30 µg), streptomycin (10 µg), gentamicin (10 µg), chloramphenicol (30 µg), clindamycin (2 µg), and erythromycin (15 µg). For enterococci, vancomycin, ampicillin, tetracycline, chloramphenicol, and erythromycin were tested at the same concentrations, with the additional inclusion of teicoplanin (30 µg), ciprofloxacin (5 µg), levofloxacin (5 µg), quinupristin/dalfopristin (15 µg), high-level streptomycin (300 µg), high-level gentamycin (120 µg), and linezolid (30 µg), to enable a more comprehensive characterization of enterococcal resistance profiles. To ensure reproducibility, 10% of isolates were randomly selected and re-tested in duplicate to confirm reproducibility of results.

#### 2.3.2. Technological Characterization

##### Survival and Growth Under Technological and Environmental Stress Conditions

The ability of isolates to adapt to cheese industry-relevant conditions was assessed by evaluating survival and growth under a range of temperature, salinity, and pH conditions. Temperature tolerance was assessed by inoculating metabolically active bacteria onto MRS agar (non-enterococcal LAB) or BHI agar (*Enterococcus* spp.) by the streak plate method, followed by incubation at 4, 10, 20, and 37 °C for 7 days [28]. Salt tolerance was evaluated by streak-plating bacteria onto MRS or BHI agar supplemented with NaCl at 1, 2.5, and 5% (*w*/*v*), followed by incubation for 24–48 h under optimal conditions [29]. In both assays, visible growth was recorded as a positive result, and absence of growth as a negative result. pH tolerance was assessed following the methodology described by Touret et al. (2018) [26]. Bacteria were grown in appropriate broth media (MRS or BHI) at 30 or 37 °C and subsequently suspended in Ringer’s solution (Oxoid, Hampshire, UK) to a final concentration of 1 × 10^6^ CFU/mL. Aliquots of 20 µL of bacterial suspension were inoculated into 96-well microplates containing 180 µL of growth medium adjusted to pH 7.0 or 8.5. Absorbance was monitored using a Tecan Sunrise™ microplate reader (Tecan Group Ltd., Männedorf, Switzerland), with data acquisition managed through xFluor4™ software (v4.51), with readings recorded at 1 h intervals over the first 6 h and additional measurements at 24 and 48 h. To ensure reproducibility, 10% of assays were performed in duplicate.

##### Milk Acidification

Ewe’s milk acidification was evaluated following the protocol described by Mrkonjic Fuka et al. (2017) [30]. Briefly, 10 mL aliquots of ewe’s milk were dispensed into sterile 15 mL screw-cap tubes for incubation, inoculated at 1% (*v*/*v*) with each metabolically active bacterium and incubated at 30 °C or 37 °C, depending on the genus. The pH was monitored at 2, 6, and 24 h of incubation using pH strips (Scharlau, Barcelona, Spain). To ensure reproducibility, 10% of isolates were randomly selected and re-tested as independent replicates, and the same batch of ewe’s milk was used for all assays.

##### Proteolytic and Lipolytic Activities

Lipase and protease production were evaluated according to previously described methods of Carlos et al., 2009 [31]. Lipase activity was assessed on Spirit Blue Agar (BD Difco, Detroit, MI, USA) supplemented with 1% (*v*/*v*) tributyrin (Thermo Fisher Scientific, Waltham, MA, USA) as substrate. Protease production was evaluated on skim milk agar, prepared with 20 g/L skim milk (Liofilchem, Roseto degli Abruzzi, Italy).

Each metabolically active bacterium was inoculated onto the respective medium using the spot-on-lawn technique and incubated under optimal growth conditions (30 or 37 °C for 24 or 48 h, depending on the isolate). Enzymatic activity was determined by the presence of clear halos and/or visible hydrolysis zones surrounding the colonies. As control, cheese isolates from *Azeitão* and *Nisa* isolated and characterized in previous years, that were lipase- and protease-positive, were used as positive controls. Ten percent of isolates were randomly selected and re-tested as independent replicates to confirm reproducibility of results.

#### 2.3.3. Inhibitory Activity of Cheese Isolates

All isolates were screened for inhibitory activity against both Gram-positive (*Listeria monocytogenes* CECT 935, *Enterococcus faecalis* ATCC 29212, *Bacillus cereus* ATCC 14579^T^) and Gram-negative (*Pseudomonas fluorescens* ATCC 13525T, *Escherichia coli* ATCC 25922) indicator strains. Cheese isolates were inoculated in agar plates (MRS for non-enterococcal LAB and BHI for *Enterococcus* spp.) by the spot-on-lawn method, described by Çadirci et al. (2005) [32], and incubated for 48 h (non-enterococcal LAB) and 24 h (*Enterococcus* spp.). Each pathogenic bacterium was suspended in sterile Ringer solution (Oxoid, Hampshire, UK) to a turbidity equivalent to 0.5 McFarland standard (≈10^8^ CFU/mL) and inoculated into semi-solid BHI agar (0.7%). The inoculated semi-solid agar was poured on top of the MRS and/or BHI spot-on-lawn plates and incubated for 24 h at 37 °C. After incubation, inhibition halos were measured, in millimeters, to assess antagonistic capacity; the absence of a halo was considered a negative result. As positive controls, cheese isolates from *Azeitão* and *Nisa* isolated and characterized in previous years, that showed inhibitory activity against all pathogenic bacteria tested, were used. In addition, 10% of isolates were randomly selected and re-tested as independent replicates to confirm reproducibility.

#### 2.3.4. Probiotic Characterization

##### Survival in the Presence of Bile Salts and Acidic pH

The capacity of the isolates to survive the gastrointestinal environment was evaluated using two complementary approaches. Survival in the presence of bile salts was assessed according to Argyri et al. (2013) [33]. Briefly, metabolically active bacteria were inoculated by the streak plate method onto agar media (MRS or BHI, depending on the isolate) supplemented with 0.5% (*w*/*v*) bile salts (Biokar Diagnostics, Beauvais, France). Plates were incubated at optimal temperatures for 24–48 h, after which the presence or absence of growth was recorded. Presence of growth was deemed a positive result, whereas no growth was a negative result. To confirm reproducibility, 10% of isolates were randomly selected and re-tested as independent replicates.

Tolerance to acidic conditions was determined following the methodology of Touret et al. (2018) [26]. Bacteria were grown in appropriate broth media (MRS or BHI) at 30 or 37 °C and subsequently suspended in Ringer’s solution (Oxoid, Hampshire, UK) to a final concentration of 1 × 10^6^ CFU/mL. Aliquots of 20 µL of bacterial suspension were inoculated into 96-well microplates containing 180 µL of growth medium adjusted to pH 2. Absorbance was monitored using a Tecan Sunrise™ microplate reader (Tecan Group Ltd., Männedorf, Switzerland), with data acquisition managed through xFluor4™ software (v4.51), with readings recorded at 1 h intervals over the first 6 h, and additional measurements at 24 and 48 h. Concurrently, to assess cell viability, spot plating was used at each measurement interval to track survival over time. All assays were performed in triplicate, encompassing three independent biological replicates and three technical replicates to ensure statistical robustness.

##### Auto- and Co-Aggregation Capabilities

Auto-aggregation and co-aggregation assays were performed according to Grujović et al. (2019) [34]. Bacterial suspensions were prepared to a final concentration of 1 × 10^9^ CFU/mL in Ringer’s solution (Oxoid, Hampshire, UK) after growth in appropriate broth media (MRS or BHI) at 30/37 °C. Assays were carried out in 96-well microplates containing 0.1 M phosphate buffer. For auto-aggregation, each well contained 198 μL of phosphate buffer and 2 μL of bacterial suspension. For co-aggregation, each well contained 196 μL of phosphate buffer, 2 μL of bacterial suspension, and 2 μL of the respective pathogenic strain suspension (*E. coli* ATCC 25922, *L. monocytogenes* CECT 935, and *Salmonella Enteritidis* CECT 4300^T^).

Absorbance was measured at 620 nm using a microplate reader (Tecan Group Ltd., Männedorf, Switzerland) at 0, 1, 2, 3, 4, 5, and 24 h. Aggregation percentage was calculated as [(A_0_ − A_t_)/A_0_] × 100, where A_0_ corresponds to the absorbance at time 0 and A_t_ to the absorbance at each time point. Isolates were classified as high, moderate, or low aggregators according to criteria previously established by Collado et al. (2008) [35].

##### Survival Under Simulated Gastrointestinal Tract Conditions

Gastrointestinal tract simulation was performed based on Campos et al. (2020) [36], with minor adaptations. Briefly, isolates were sequentially exposed to oral, gastric, intestinal, and large-intestine simulated conditions. First, bacterial suspensions were prepared to a final concentration of 1 × $10^{9}$ CFU/mL in Ringer’s solution after growth in appropriate broth media (MRS or BHI) at 30/37 °C. The oral phase consisted of incubation in Ringer solution (pH 6.5) supplemented with 150 mg/L lysozyme (Merck, Germany) at 37 °C for 5 min (50 rpm). After centrifugation (10,000 *g* for 5 min), the gastric phase was simulated using Ringer solution supplemented with 3 g/L pepsin (Merck, Germany), first at pH 3.0 (Stomach 1) and then at pH 2.0 (Stomach 2), each for 45 min at 37 °C and 100 rpm, with an intermediate centrifugation step (10,000 *g* for 5 min). The intestinal phase was simulated in Ringer solution (pH 6.5) supplemented with 3 g/L bile salts (Biokar Diagnostics, France) and 1 g/L pancreatin (U.S.P, The Wilson laboratories), followed by incubation for 2 h at 37 °C and 100 rpm (Intestine 1). Finally, the large-intestine phase was mimicked by incubation in Ringer solution (pH 7.0) for 1 h at 37 °C and 100 rpm (Intestine 2).

Viability was assessed at each stage by spread plating serial dilutions onto MRS agar (non-enterococcal LAB) and BHI agar (*Enterococcus* spp.). Samples were collected before each centrifugation step. The results were expressed as the mean log counts and plotted over time to characterize the survival kinetics of each isolate under gastrointestinal tract conditions. *Limosilactobacillus reuteri* ATCC 6475, a commercial strain, was used as a positive control strain due to its extensively documented GIT resilience profile, which provides a reproducible methodological benchmark and confirms the biological stringency of the simulation model. Its inclusion is not intended to imply functional equivalence between the food-derived isolates and commercially developed probiotic preparations; rather, it contextualizes the survival data within an established reference framework consistent with current in vitro probiotic characterization practices [37].

### 2.4. Statistical Analysis

All statistical analyses were performed using RStudio (version 4.4.0) utilizing the *tidyverse* v2.0.0 [38], *readxl *v1.4.3 [39], *rstatix* v0.7.2 [40], and *ggpubr* v0.6.0 [41] packages. Prior to modeling, microbiological enumeration counts were transformed to log10 values to stabilize variance and satisfy parametric assumptions. For all statistical tests, significance was established at α = 0.05.

#### 2.4.1. Bacterial Enumeration and Production Stability Trend

The effects of culture medium (M17, MRS, SBA), region (*Azeitão*, *Nisa*), year (2021, 2022), and their interactions on log10-transformed microbial counts were assessed. Prior to analysis, the assumptions of normality of residuals and homogeneity of variance were evaluated using the Shapiro–Wilk test and Levene’s test, respectively. If both assumptions failed, a non-parametric Aligned Rank Transform (ART) ANOVA was applied instead, using the ARTool package (version 0.11.2). The ART procedure aligns and rank-transforms the response variable separately for each model term, enabling non-parametric factorial analysis with interaction effects. A full factorial model including all main effects and two-way and three-way interactions was fitted. Post hoc pairwise comparisons were conducted for significant terms (*p* < 0.05) using the emmeans package, with Bonferroni correction for multiple testing. Statistical significance was set at α = 0.05.

#### 2.4.2. Cellular Aggregation Dynamics

Optical density (OD) at 600 nm absorbance values, obtained from three technical replicates per isolate per time point, were used to calculate the percentage of aggregation according to the following formula in Section Survival under Simulated Gastrointestinal Tract Conditions. To assess differences in aggregation percentages across incubation periods, a one-way repeated measures analysis of variance (RM-ANOVA) was performed per isolate for each specific assay (auto-aggregation, and co-aggregation with *E. coli*, *L. monocytogenes*, and *S. enteritidis*), using the incubation period as the within-subject factor and technical replicates as the subject identifier; this repeated-measures design was intended to characterize the aggregation kinetics of each isolate individually and was not used to draw statistical comparisons between isolates, avoiding pseudoreplication in cross-isolate inference. Prior to each test, the assumption of normality was evaluated using the Shapiro–Wilk test applied to the aggregation values at each time point. When normality could not be assumed, the non-parametric Friedman test was applied as an alternative. For all significant overall tests, pairwise post hoc comparisons between time points (t4 vs. t5, t4 vs. t24, and t5 vs. t24) were conducted using paired t-tests or Wilcoxon signed-rank tests, as appropriate. All post hoc *p*-values were adjusted for multiple comparisons using the Benjamini–Hochberg FDR correction. Results were visualized as bar charts displaying mean percentage aggregation ± standard deviation for each incubation period, faceted by isolate using ggplot2 [41].

For all assays, statistical significance thresholds were visually annotated on figures as follows: * *p* <0.05, ** *p* < 0.01, *** *p* < 0.001, and ns (not significant).

#### 2.4.3. Survival in Simulated Gastrointestinal Tract Conditions

Colony-forming unit counts (CFU/mL) obtained from three independent biological replicates, each evaluated in triplicate (*n* = 9 observations per group), were used to assess bacterial survival across simulated GIT conditions via two complementary approaches. First, to evaluate cumulative bacterial decay, log10 CFU/mL values of the initial and final suspensions were compared for each isolate independently using a Welch two-sample *t*-test. Second, to assess the performance of each isolate relative to the reference control strain under each simulated GIT compartment (Mouth, Stomach 1, Stomach 2, Intestine 1, and Intestine 2), Welch two-sample *t*-tests were executed for each isolate–condition pair. The Welch t-test was selected over Student’s *t*-test because it does not assume equal variances between groups. To control the false discovery rate (FDR) arising from multiple comparisons, raw *p*-values were adjusted using the Benjamini–Hochberg (BH) procedure. Correction was applied across the four isolate comparisons for the decay analysis, and across all 15 pairwise tests (three isolates × five conditions) for the isolate-versus-control analysis. Results were visualized as grouped bar charts generated via ggplot2 v3.5.1 displaying mean log10 CFU/mL ± standard deviation for each condition, with significance brackets indicating pairwise differences between each isolate and the control strain.

## 3. Results

The results are presented according to the polyphasic strategy adopted to document, preserve, and characterize the indigenous microbiota of *Azeitão* and *Nisa* PDO cheeses. This workflow comprised the establishment of a representative collection of autochthonous isolates, followed by the progressive selection and characterization of lactic acid bacteria (LAB) based on safety, technological performance, and probiotic-related traits. This approach allowed the identification of functionally relevant isolates while generating a curated microbial repository representative of the biodiversity associated with these Portuguese traditional cheeses.

### 3.1. LAB Enumeration and Isolation

**Figure 1:**
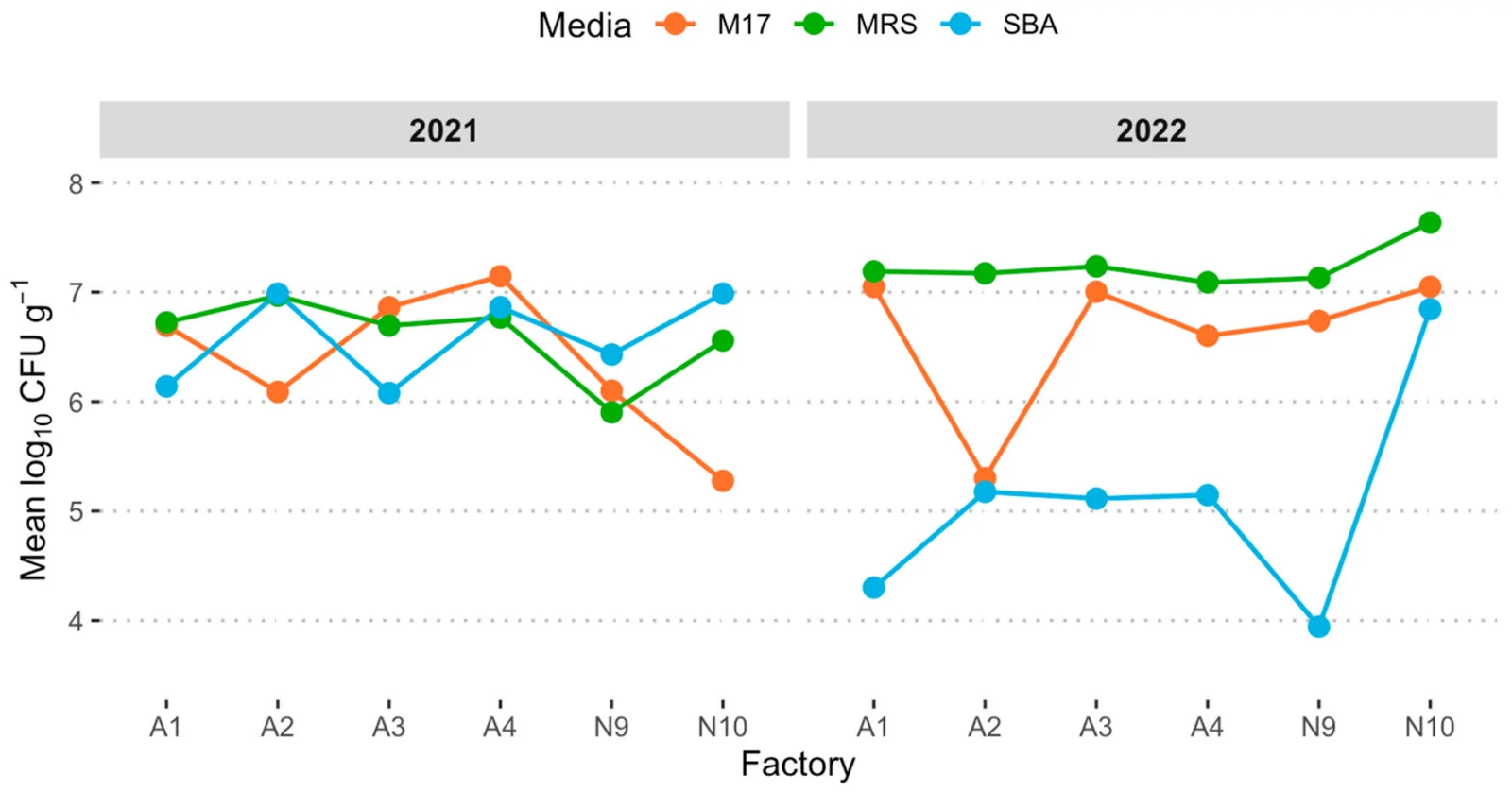
Variability of bacterial enumeration in PDO cheeses produced in Azeitão and Nisa throughout two production years. Cheese factories from Azeitão are A1–A4; Cheese factories from Nisa are N9–N10. The media used are M17 in orange; MRS—Man, Rogosa and Sharpe agar in green; SBA—Slanetz and Bartley agar in blue.

The longitudinal analysis of microbial counts across 2 years of production (2021–2022) revealed that culture medium was the primary factor influencing bacterial recovery, though regional influences were also evident. Microbial counts for the six producers, four from *Azeitão* (A1–A4) and two from *Nisa* (N9–N10), are summarized in Figure 1 and Supplementary Table S1. Throughout 2021 and 2022, there were changes in log10 counts between cheese factories from the same region. Even so, MRS and M17 media had overall the highest counts in *Azeitão* apart from A2, where SBA counts appeared to be higher compared to those in M17 (10^6^ to 10^7^ CFU/g). In the same year, *Nisa* SBA medium showed higher counts than MRS and M17. In 2022, more uniform CFU/g counts were apparent in the case of MRS, which was the medium with higher counts. M17 also maintained high counts, except for A2, where there was an abrupt decrease. SBA showed lower counts, especially for A1 and N9, with 10^4^ CFU/g in both cases.

Statistical analysis was performed to assess if there were statistical changes between media, regions and production years. Since normality was not achieved (evaluated using Shapiro–Wilks and Levene’s test), a non-parametric approach was used, the Aligned Rank transformation ANOVA. When comparing media vs. year in 2021, there were significant differences between MRS and SBA (*p* = 0.0044), where MRS had significantly higher counts than SBA; and M17 and SBA (*p* = 0.0205), where M17 also had higher counts than SBA. No statistical differences were found between MRS and M17. For 2022, no statistical differences were found between media. Another analysis was performed to assess if there were statistical differences between the 2 years in microbial counts; in this case, no statistical differences were found in M17, whereas in MRS (*p* = 0.007) and SBA (*p* = 0.006), differences were found. These results indicate that viable bacterial counts differed according to production year; as counts alone do not capture compositional diversity, this pattern is interpreted with caution.

### 3.2. Diversity Evaluation and Taxonomic Assignment

Approximately 20% of the colonies recovered from each isolation plate were randomly selected from each culture medium, resulting in a collection of 2304 isolates. This repository was established to preserve the culturable autochthonous microbiota associated with *Azeitão* and *Nisa* PDO cheeses produced during 2021 and 2022, creating a microbial heritage collection that safeguards the indigenous bacterial diversity underpinning the terroir and authenticity of these traditional products.

Following the establishment of the microbial collection, genetic diversity was assessed by RAPD-PCR to identify representative, genomically distinct LAB isolates for subsequent functional characterization. Fingerprinting, using the (GTG)_5_ and OPC15 primers, enabled the clustering of genetically related isolates. A representative subset was then selected from each cluster to consolidate the collection, while preserving its ecological and temporal diversity. Particular emphasis was placed on ensuring the representation of isolates from all sampled dairies and both production years. This strategy reduced the initial collection of 2304 isolates to a curated core set of 119 representative LAB, which were subsequently identified at the genus and species levels by PCR. Isolates displaying identical or highly similar RAPD-PCR fingerprints within the same producer and sampling year were considered putative duplicates, and only one representative isolate per cluster was retained; redundant (duplicate) isolates were therefore excluded prior to the functional characterization described below. The taxonomic distribution of these isolates is presented in Table 1.

The primer pair LbLMA1-rev/R16-1 targets the 16S–23S rRNA intergenic spacer region of the former *Lactobacillus* genus. As these primers were developed prior to the 2020 taxonomic reclassification of the family *Lactobacillaceae* [41], they do not discriminate among the newly established genera. Consequently, isolates yielding amplification with the general primer pair but not with any of the species-specific primers were conservatively assigned as *Lactobacillaceae* sp.

Overall, among the 119 LAB isolates, the most prevalent genus was *Enterococcus* sp., 65 isolates (55%), followed by *Lactobacillaceae* sp., 28 isolates (24%), *Leuconostoc* sp., 24 (21%), and one each of *Pediococcus* sp. and *Lacticaseibacillus*. At the species level, 48 isolates were identified as *E. faecalis*, two as *E. faecium*, 9 as *E. durans*, and one as *E. hirae*. Among the remaining LAB, seven were classified as *L. mesenteroides*, four as *Lactiplantibacillus plantarum*, and one as *Lacticaseibacillus paracasei*. Forty-six isolates (24 *Lactobacillus* sp., 17 *Leuconostoc* sp., and five *Enterococcus* sp.) could not be identified at the species level.

### 3.3. Safety Evaluation

Following the establishment of a representative microbial collection encompassing the culturable autochthonous diversity of *Azeitão* and *Nisa* PDO cheeses produced during 2021 and 2022, the selected isolates were functionally characterized to explore their potential for future food and probiotic applications. As a first step, a comprehensive safety assessment was performed to ensure their suitability for such applications. This included the evaluation of hemolytic and gelatinolytic activities at both phenotypic and genotypic levels, followed by antimicrobial susceptibility testing. Isolates exhibiting traits associated with pathogenicity or other safety concerns were systematically excluded from further analyses, ensuring that only safe candidates were retained for technological and probiotic characterization. The complete safety assessment is presented in Supplementary Table S2.

#### 3.3.1. Hemolytic and Gelatinolytic Activities: Phenotypic and Genotypic Analysis

Hemolysis and gelatinase production are key virulence traits frequently monitored in *Enterococcus* species, as they are associated with the breakdown of host tissues and potential pathogenicity [22]. Consequentially, hemolytic activity was assessed for all 119 isolates (enterococcal and non-enterococcal LAB) on horse blood agar under anaerobic incubation. These conditions were selected to minimize external oxidative factors that could interfere with erythrocyte stability while simultaneously mimicking the anaerobic environment of the human gastrointestinal tract [22]. Among non-enterococcal LAB, 55% were considered gamma hemolytic, and 45% alpha hemolytic. Among the enterococci, no isolates were beta hemolytic, most were gamma hemolytic (68%), and 32% displayed alpha hemolysis.

Regarding gelatinolytic activity, the production of gelatinolytic enzymes has been associated with a virulence trait in *Enterococcus*, due to the ability to hydrolyze gelatin and collagen in the host, among other protein substrates [42]. Gelatinase activity was tested for all isolates, both *Enterococcus* and non-enterococcal LAB; however, only positive isolates were found among *Enterococcus*, where 15 isolates, 13 *E. faecalis* (A4.7.2021, A4.21.2021, N10.49.2021, A1.14.2022, A2.34.2022, A4.5.2022, A4.12.2022, A4.30.2022, N9.1.2022, N9.23.2022, N9.58.2022, N10.17.2021, A2.40.2022), one *Enterococcus* sp. (N9.26.2021) and one *E. durans* (A4.11.2022) showed ability to degrade gelatin.

Simultaneously with the phenotypic evaluation, a screening for virulence determinants was also performed, but only for *Enterococcus* isolates. Among the most described virulence determinants in enterococci are two adhesins, aggregation substance (*agg*) and enterococcal surface protein (*esp*), and two factors involved in tissue destruction: gelatinase (*gel*E) and cytolysin (*cylA*). Among the complete *Enterococcus* collection (*n* = 65), 29 were negative for all virulence determinants studied by multiplex-PCR. As for the remainder, among the tested adhesins, the *agg* gene was present in three *E. faecalis* isolates. Additionally, four isolates were positive for the *esp* gene, specifically two *E. faecalis*, one *E. durans*, and one *Enterococcus* sp. As for *gel*E and *cyl*A factors, 35 isolates were positive for *gel*E; among them, 33 were *E. faecalis*, and the remainder were *E. durans* and *Enterococcus* sp. In the case of *cyl*A, three isolates were positive, among them one *E. faecalis*, one *E. durans* and one *Enterococcus* sp. Overall, the most prevalent gene identified was *gel*E, with a prevalence of around 41%. In addition, in some cases, a combination of virulence genes was observed. Eight isolates showed the presence of more than one virulence factor, A4.11.2022 (*E. durans*) was positive for *gel*E, *cyl*A and *esp*; A1.51.2022, N9.23.2022 and N9.58.2022 (all *E. faecalis*) were positive for *gel*E and *agg*; N10.18.2022 (*E. faecalis*) was positive for *gel*E and *cyl*A; A4.18.2021 (*Enterococcus* sp.) N10.17.2021 and N10.58.2021 (*E. faecalis*) were positive for *gel*E and *esp*.

#### 3.3.2. Antimicrobial Susceptibility Test

Antimicrobial susceptibility testing was performed separately for each taxonomic group: *Enterococcus* (*n* = 65 isolates) and other LAB (*n* = 54). This separation accounts for the different interpretive criteria and safety implications required for the assessment of potentially pathogenic *versus* traditionally safe food-grade bacteria. Therefore, using the disk diffusion method, susceptibility testing of all 119 isolates was performed against 14 antimicrobials. The results were interpreted according to the microbiological breakpoints defined by Charteris et al. (1998) [25] and Touret et al. (2018) [26] for non-enterococcal LAB, and the Clinical and Laboratory Standards Institute (CLSI) guidelines M100 [27] for enterococci.

Some differences between breakpoint criteria were found when evaluating non-enterococcal LAB resistance, the most important one being the breakpoint for the antibiotic ampicillin, where, in Touret’s study, the breakpoint was >37 mm considered to be susceptible, whereas in Charteris, the breakpoint was >12 mm. Given this discrepancy, the resistance profile of the isolates was analyzed according to Touret et al. (2018) criteria [26].

The highest resistance frequencies were recorded for vancomycin (*n* = 48 resistant isolates), followed by kanamycin (*n* = 27 resistant isolates), streptomycin (*n* = 27 resistant isolates) and gentamycin (*n* = 22 resistant isolates). All isolates (*n* = 54) were classified as susceptible to clindamycin and erythromycin, and two isolates, A3.22.2021 and A3.15.2022 (both *Leuconostoc* sp.), were classified as resistant to tetracycline. For the antibiotic ampicillin, 24 isolates were classified as resistant (10 *Lactobacillaceae* sp., nine *Leuconostoc* sp., three *L. mesenteroides*, one *Lactiplantibacillus plantarum* and one *Lacticaseibacillus paracasei*). Similarly, although lower in number, against the antibiotic chloramphenicol, two isolates, A4.46.2021 (*Lactobacillaceae* sp.) and A3.59.2022 (*Leuconostoc* sp.), were classified as resistant.

In the case of *Enterococcus,* the highest frequency of resistance was observed for quinupristin-dalfopristin (*n* = 47 resistant isolates) (considered intrinsic for *E. faecalis* [43]), followed by tetracycline (*n* = 38 resistant isolates) and teicoplanin (*n* = 28 resistant isolates). Lower resistances were recorded for erythromycin and ciprofloxacin (both *n* = 12 resistant isolates), chloramphenicol (*n* = 10 resistant isolates), vancomycin (*n* = 8 resistant isolates) ampicillin (*n* = 2 resistant isolate) and levofloxacin (*n* = 1 resistant isolate). Considering that low-level resistance to streptomycin and gentamicin is often considered intrinsic in the *Enterococcus* genus, this study used high-level concentrations to screen for acquired resistance; consequently, only 13 isolates were identified as resistant to streptomycin and none to gentamicin. Similarly, all isolates showed susceptibility to linezolid.

The complete safety screening enabled the selection of a subset of isolates for subsequent technological and probiotic assessment. Isolates were excluded based on the following criteria: phenotypic expression of hemolytic or gelatinolytic activity; presence of virulence determinants, whether single or multiple; and carriage of suspected transferable antibiotic resistance determinants. Isolates meeting any of these criteria were considered potentially pathogenic and excluded from further studies (full safety characterization results are provided in Supplementary Tables S2 and S3).

Among non-enterococcal LAB, 26 isolates were excluded due to safety concerns, comprising *Lactobacillaceae* sp. (*n* = 10), *L. mesenteroides* (*n* = 3), *Leuconostoc* sp. (*n* = 11), *Lactiplantibacillus plantarum* (*n* = 1) and *Lacticaseibacillus paracasei* (*n* = 1). A more substantial reduction was required within the *Enterococcus* collection, where 52 isolates were excluded, primarily *E. faecalis* (*n* = 44), followed by *Enterococcus* sp. (*n* = 4), *E. durans* (*n* = 3), and *E. faecium* (*n* = 1). In total, 29 non-enterococcal LAB and 10 *Enterococcus* isolates met all safety criteria and proceeded to the technological evaluation stage.

### 3.4. Survival and Growth Under Technological and Environmental Stress Conditions

Following safety screening, the selected isolates were qualitatively evaluated for their ability to survive and grow under conditions relevant to both dairy processing and the gastrointestinal environment. Such resilience is a fundamental prerequisite for strain selection, as LAB intended for use as starter, adjunct, or probiotic cultures must remain viable throughout food production and, where applicable, withstand gastrointestinal transit while retaining the functional properties required for technological performance or probiotic activity. To this end, isolates were first tested for their ability to grow at various temperatures (4, 10, 20, and 37 °C) and under salt-induced osmotic stress (1, 2.5, and 5% NaCl). With respect to thermal tolerance, in the non-enterococcal LAB (*n* = 29) collection, 9 isolates (A1.50.2021, A3.54.2021, A3.37.2022, A3.61.2022, N9.1.2022, N9.29.2022, N9.49.2022, all *Lactobacillaceae* sp., and A4.41.2022 and A1.64.2021, both *L. mesenteroides*) showed no growth at 4 °C. Among these isolates, A1.50.2021, A3.37.2022, A3.61.2022, A4.41.2022 and N9.49.2022 also did not grow at 10 °C, as well as A2.6.2022, A3.55.2021 and N9.3.2021 (all *Lactobacillaceae* sp.). At 20 °C, all isolates were able to grow; however, A2.12.2021 and A2.29.2021 (both *L. mesenteroides*) did not grow at 37 °C. In the case of *Enterococcus*, although lower in number, these isolates showed better adaptation to changes in temperature since only two isolates, A1.45.2021 (*E. durans*) and A3.4.2021 (*E. faecalis*), showed no growth at 4 °C. Similarly, when NaCl concentrations were tested, all isolates were able to grow throughout all percentages tested. In the case of non-enterococcal LAB, five isolates, A4.8.2022 (*L. mesenteroides*), A2.12.2021, A2.29.2021, A2.36.2021 (all *Leuconostoc* sp.) and N9.10.2022 (*Lactiplantibacillus plantarum*) did not grow when tested with 5% NaCl. Complete technological assessment results are provided in Supplementary Tables S3 and S4.

Building upon this environmental resilience, the metabolic potential of the collection was further evaluated through acidification kinetics in ewe’s milk and enzymatic activity assays. All isolates showed the capacity to reduce the pH of sheep’s milk over a 24 h period. After 2 h of incubation, all isolates had either reduced pH by at least one unit or maintained it within pH 7.0 ± 0.5. By 6 h, the majority had reduced pH to 6.0 ± 0.5; an exception was isolate A4.20.2022 (*E. durans*), which achieved a more rapid acidification to pH 5.0 ± 0.5 at this time point. In contrast, the isolates *Lactobacillaceae* sp., *Leuconostoc* sp. (including *L. mesenteroides*) and *Pediococcus* p. displayed slower initial kinetics, remaining at or above pH 6.0 ± 0.5 at 6 h. By 24 h, however, non-enterococcal LAB achieved the most pronounced total acidification, reaching pH 4.0 ± 0.5, particularly *Lactobacillaceae* sp. (A1.50.2021, N9.49.2022), *Lactiplantibacillus plantarum* (N9.10.2022, N9.58.2022), and *L. mesenteroides* (A4.42.2022). Among *Enterococcus*, pH stabilized around 5.0 ± 0.5 by the end of the incubation period.

Complementing these acidification profiles, proteolytic and lipolytic activities, both critical for flavor development in cheese, varied considerably across genera. In non-enterococcal LAB, only four isolates showed proteolytic activity, among them A3.54.2021, A3.55.2021 (both *Lactobacillaceae* sp.), A2.12.2021 (*Leuconostoc* sp.) and A2.24.2021 (*Lactiplantibacillus plantarum*). In the *Enterococcus* collection, only A3.4.2021 (*E. faecalis*) was positive for proteolytic activity. A higher number of isolates showed lipolytic activity; among non-enterococcal LAB, over 16 isolates were positive, including seven Lactobacillaeceae sp., two *L. mesenteroides*, four *Leuconostoc* sp., two *Lactiplantibacillus plantarum* and one *Pediococcus* sp.

### 3.5. Inhibitory Activity Against Pathogenic and Deteriorative Strains

The inhibitory activity of the selected isolates against foodborne pathogens and spoilage microorganisms was also evaluated, providing further evidence of their potential as both technological cultures and probiotic candidates. The following indicator bacteria were used: three Gram-positive bacteria, *E. faecalis*, *B. cereus* and *L. monocytogenes*; and two Gram-negative bacteria, *E. coli* and *P. fluorescens*. Non enterococcal LAB (*n* = 29) showed higher inhibitory activity against *E. faecalis* (*n* = 17), followed by *E. coli* (*n* = 16), *L. monocytogenes* and *P. fluorescens* (*n* = 12 in both cases). Notably, seven *Lactobacillaceae* sp. (A1.50.2021, A2.06.2022, A2.30.2022, N9.3.2021, N9.1.2022, N9.29.2022, N9.49.2022) and one *L. mesenteroides* (A4.8.2022) inhibited all indicator bacteria. In the *Enterococcus* (*n* = 10) collection, higher inhibitory activity was observed for *L. monocytogenes*, where seven out of 10 isolates were able to inhibit this bacterium. Against *E. faecalis*, five isolates showed an inhibitory effect, followed by *E. coli* (*n* = 3) and *P. fluorescens* (*n* = 2). Similarly to LAB, two isolates showed inhibitory activity against all tested indicator bacteria, one *E. faecalis* (A1.55.2022) and one *E. hirae* (A1.8.2022).

### 3.6. Characterization of Probiotic Properties

Beyond their well-established role in cheese ripening and flavor development, LAB may enhance both the safety and functional value of fermented foods. Depending on the strain, they can inhibit undesirable microorganisms and exert health-promoting effects, including modulation of the gut microbiota, immunomodulatory activity, reinforcement of intestinal barrier function, and the sequestration or biotransformation of certain toxic compounds [44].

Bile salts are essential constituents of the gastrointestinal environment, possessing detergent-like properties that can disrupt bacterial lipid membranes. Consequently, the ability to tolerate these salts is a fundamental selection criterion for probiotic candidates, as it ensures their survival and functional viability during passage through the intestinal tract [45]. In addition to enzymatic degradation, the low pH levels (2.0–3.0) typical of the gastric phase represent a significant barrier to bacterial survival [46]. Therefore, evaluating acid tolerance is crucial to ensure that cheese-isolated strains can survive the stomach’s acidic environment and successfully colonize the gut [46]. After the technological characterization, the first assessment of the LAB strains, regarding their probiotic potential, concerned the ability to multiply in the presence of bile salts, as well as the tolerance to pH (2, 7 and 8.5). Following these assays, a further selection step was applied, retaining only isolates that demonstrated resistance to bile salts and acidic pH, key prerequisites for survival through the gastrointestinal tract. Auto- and co-aggregation capabilities were subsequently assessed in the selected candidates.

#### 3.6.1. Resistance to Bile Salts and Acidic pH

The pH and bile assays were used as preliminary tests for selecting LAB isolates to undergo a comprehensive gastrointestinal tract experiment aimed at identifying potential probiotic candidates. Resistance to bile salts was evaluated using growth medium MRS (non-enterococcal LAB) or BHI (*Enterococcus*) supplemented with 0.5% of bile salts. Among non-enterococcal LAB (*n* = 29), most isolates were able to grow in the presence of bile (*n* = 23); isolates that showed no growth included five from *Lactobacillaceae* sp. (A3.54.2021, A3.37.2022, A3.61.2022, N9.29.2022, N9.49.2022) and one *Leuconostoc* sp. (A2.29.2021). In the *Enterococcus* (*n* = 10) collection, all isolates were able to grow in the presence of bile. As for resistance to pH, aside from acidic pH, pH levels of 7.5 and 8 were also tested. All isolates demonstrated the ability to grow at pH 7.5 and 8. The survival at pH 2 was assessed over a 6 h incubation period. As time progressed, the survival rate of all isolates decreased; among non-enterococcal LAB, 14 isolates remained viable after the first hour of incubation; at 2 h, only seven, and at 3 h, only two remained viable. Regarding *Enterococcus*, after the first hour of incubation, seven isolates remained viable; at 2 h, four, and at 3h, the same four maintained their viability. By 6 h, in both collections, no isolate was able to continue multiplying.

After performing both assays, a new selection step was applied: only isolates that were able to survive between 1 and 2 h under acidic pH and resistant to bile salts were selected. This categorical bile-salt and pH screening was used deliberately as a rapid, industry-standard preliminary filter to progressively narrow a large candidate pool (*n* = 119) prior to the fully quantitative simulated gastrointestinal tract assay, which does provide isolate-level survival data as viable counts over time (Section 3.6.3); this selection reduced the microbial collection under characterization to 10 isolates: two *L. mesenteroides* (A2.36.2021, A1.64.2021), three *Lactobacillaceae* sp. (A3.54.2021, N9.3.2021, N9.1.2022), three *E. faecalis* (A3.4.2021, A3.47.2022, A1.55.2022), one *E. faecium* (A2.32.2021), and one *Enterococcus* sp. (A1.8.2022). These isolates were tested in parallel regarding their auto-aggregation and co-aggregation capabilities as well as survival under simulated gastrointestinal tract conditions.

#### 3.6.2. Auto-Aggregation and Co-Aggregation

**Figure 2:**
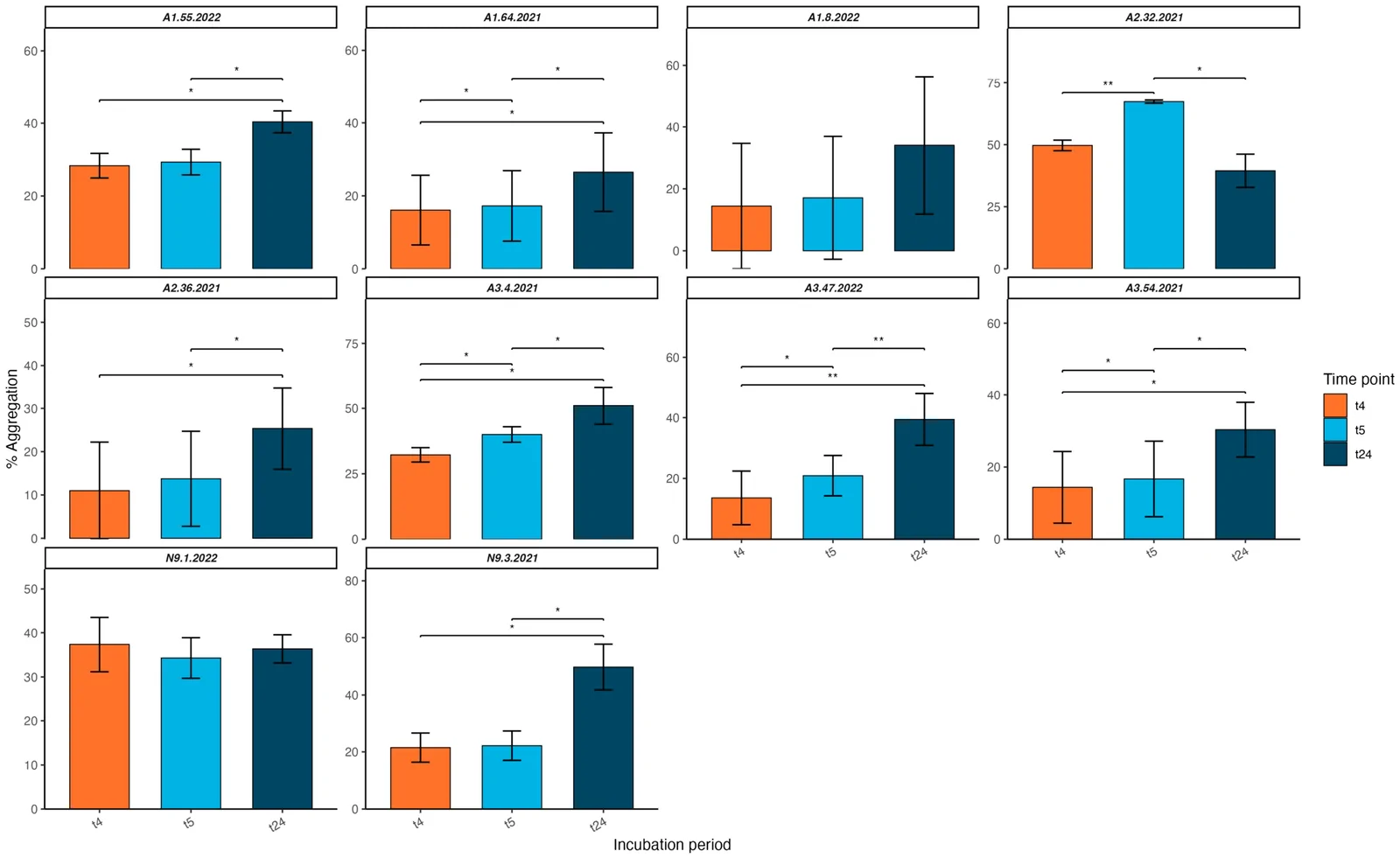
Auto-aggregation of selected isolates (*n* = 10) over time. Bar charts represent the mean percentage of auto-aggregation ± standard deviation (*n* = 3 technical replicates) for each isolate at three incubation periods (t4, t5, and t24 h). Aggregation percentages were calculated from OD_600_ absorbance readings according to the formula: % aggregation = ((OD_t0 − OD_tx)/OD_t0) × 100, where OD_t0 represents the mean absorbance prior to incubation. Significance brackets indicate adjusted *p*-values between time points for each isolate: * *p* < 0.05, ** *p* < 0.01. Absence of brackets indicates no statistically significant difference between time points for that isolate.

**Figure 3:**
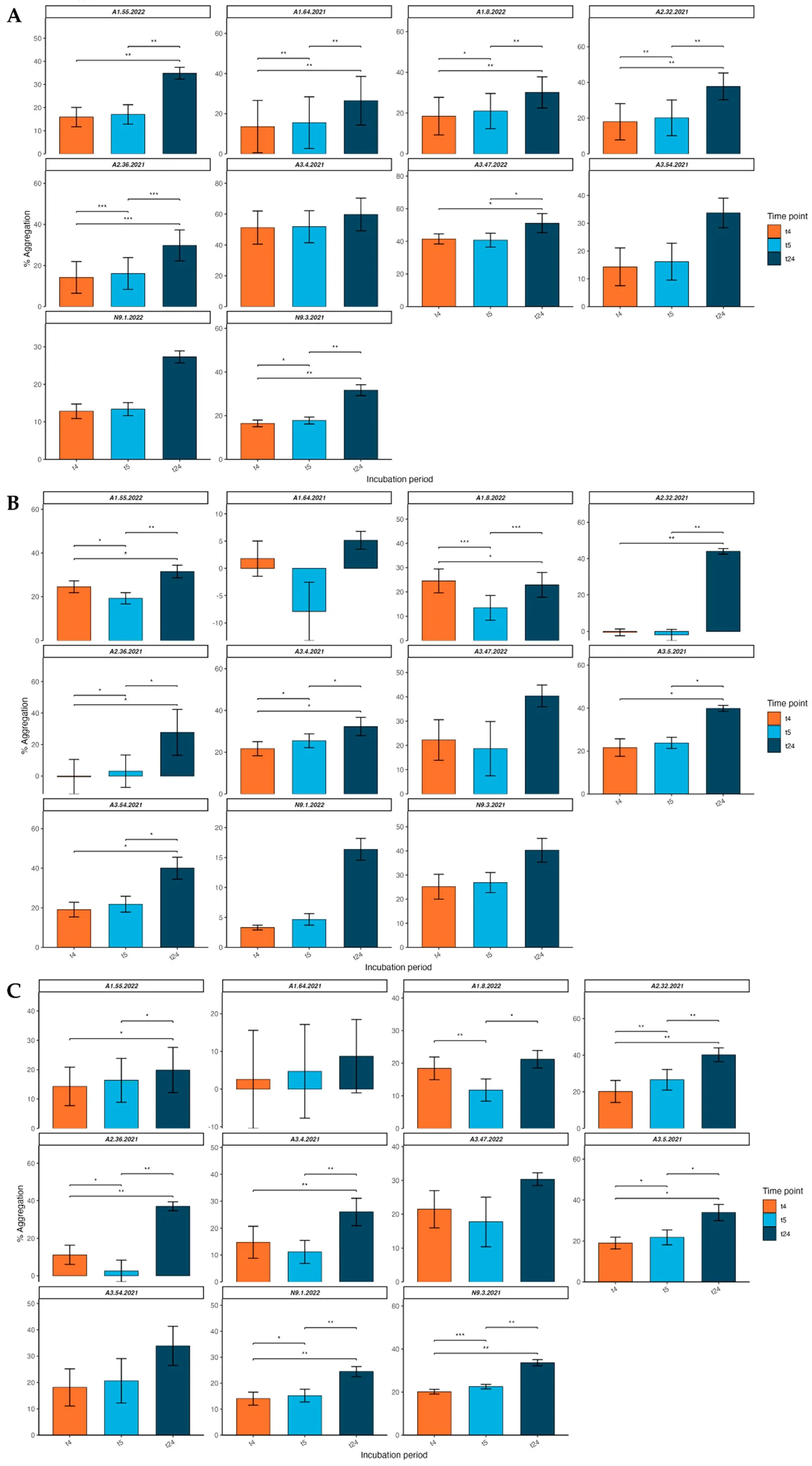
Co-aggregation of lactic acid bacteria isolates over time with *E. coli* (**A**), *L. monocytogenes* (**B**) and *S. enteritidis* (**C**). Bar charts represent the mean percentage of co-aggregation ± standard deviation (*n* = 3 technical replicates) for each isolate at three incubation periods (t4, t5, and t24 h). Significance brackets indicate adjusted *p*-values between time points for each isolate: * *p* < 0.05, ** *p* < 0.01, *** *p* < 0.001. Absence of brackets indicates no statistically significant difference between time points for that isolate.

The results for the auto-aggregation and co-aggregation assays are summarized in Figure 2 and Figure 3A–C, respectively, and those for the statistical analysis are listed in Supplementary Table S7. Isolates were categorized based on their aggregation percentages as low (auto-aggregation < 30%; co-aggregation < 20%), moderate (auto-aggregation 30–50%; co-aggregation 20–40%), or high (auto-aggregation ≥ 50%; co-aggregation ≥ 40%) [35,47].

Auto-aggregation capacity varied among isolates and generally increased over the incubation period (Figure 2). Among the ones with the highest auto-aggregation capabilities were A2.32.2021 (*E. faecium*), which, at between 4 h and 5 h of incubation, showed high auto-aggregation (percentages between 54 and 64%). A3.54.2021, A1.8.2022, A3.4.2021, A3.47.2022, A1.55.2022 and N9.3.2021 were considered as moderate auto-aggregators since the highest aggregation was between 30 and 50%. The remaining isolates, A1.64.2021 and A2.36.2021, were classified as low auto-aggregators since their aggregation percentages remained below 30% even after 24 h of incubation. Statistical differences were found mainly between t4–t24 (*p* < 0.05) and, in A3.54.2021, t4–t5; A3.47.2022, t4–t24 and t5–t24 (*p* < 0.01) (Figure 2 and Supplementary Table S7).

Co-aggregation also varied among indicator bacteria. Even so, as time passed, an increasing trend in co-aggregation was observed. High co-aggregation with *E. coli* was observed with two isolates, A3.4.2021 and A3.47.2022, with percentages of aggregation in the ranges of 51–60% and 41–51%, respectively. The remaining isolates were considered moderate aggregators since their percentages of aggregation were between 26 and 35%. Co-aggregation with *L. monocytogenes* identified one high aggregator, A2.32.2021; however, this isolate only had measurable OD at 24 h of incubation, appearing to require longer periods of incubation to show aggregation with *L. monocytogenes*. Six isolates, A3.4.2021, A3.47.2022, A1.8.2022, A1.55.2022, A3.54.2021 and N9.3.2021, were classified as moderate aggregators. N9.1.2022 and A1.64.2021 were classified as low aggregators. A2.36.2021 showed a co-aggregation of 25%, however, only at 24 h of incubation; therefore, it should also be considered a moderate aggregator. Finally, co-aggregation with *S. enteritidis* showed lower aggregations when compared to the remaining indicator bacteria (*L. monocytogenes* and *E. coli*). Still, all isolates except for A1.64.2021 were classified as moderate co-aggregators. Similarly to auto-aggregation, most statistical differences were found between t4 and t24 h of incubation.

#### 3.6.3. Survival Under Simulated Gastrointestinal Tract Conditions

**Figure 4:**
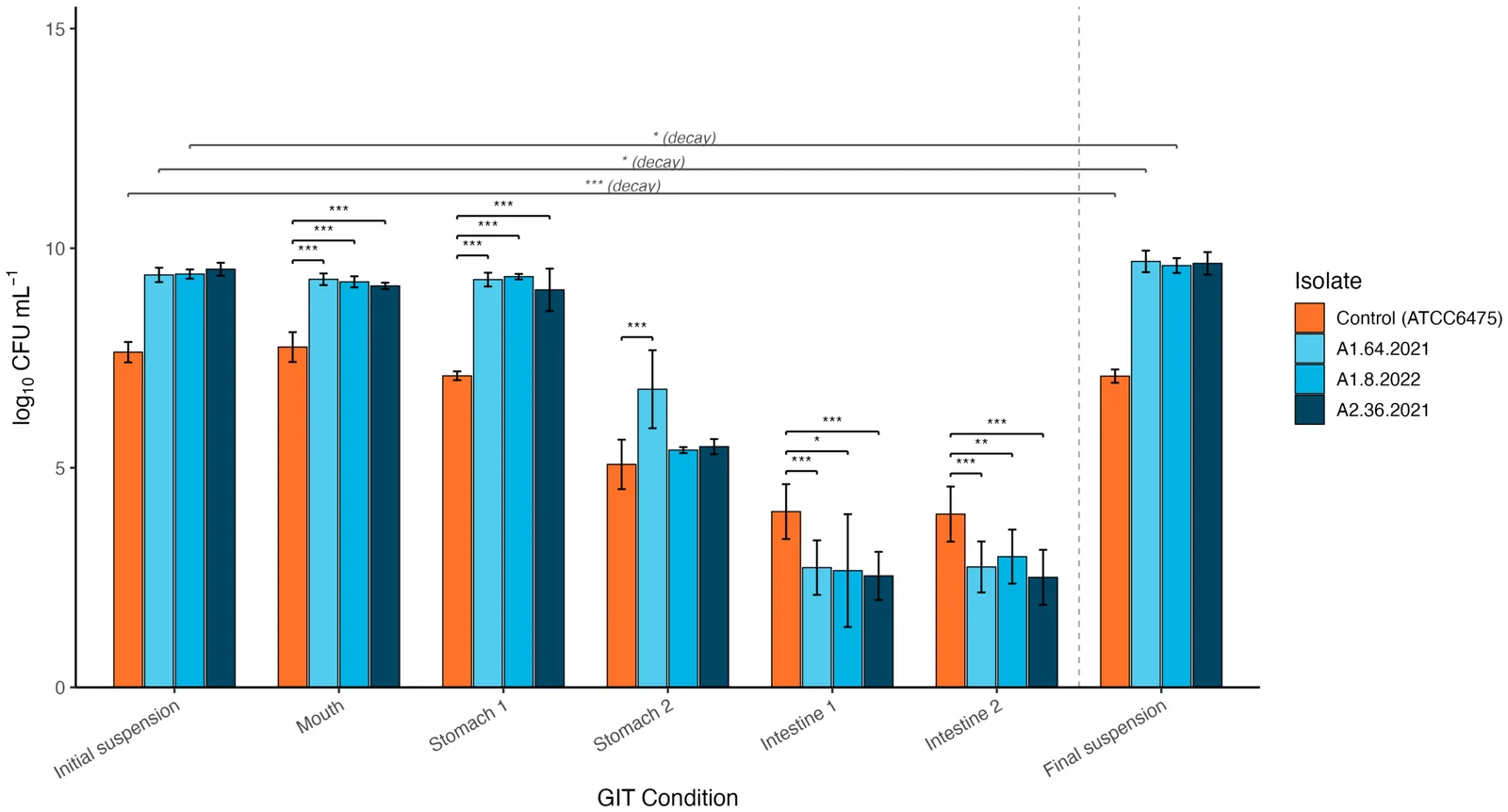
Viability of LAB isolates (colors in shades of blue) and the reference strain, Limosilactobacillus reuteri ATCC 6475 (orange), during simulated gastrointestinal tract (GIT) transit. Results are expressed as Mean Log_10_ CFU/mL + Standard Deviation. The “Initial” and “Final” suspensions represent the control for natural bacterial decay throughout the assay period. Significance brackets indicate adjusted *p*-values: * *p* < 0.05, ** *p* < 0.01, *** *p* < 0.001.

The 10 selected isolates, comprising two *Leuconostoc* sp. (A2.36.2021, A1.64.2021), three *Lactobacillaceae* sp. (A3.54.2021, N9.3.2021, N9.1.2022), three *E. faecalis* (A3.4.2021, A3.47.2022, A1.55.2022), one *E. faecium* (A2.32.2021), and one *E. hirae* (A1.8.2022), were tested for survival under simulated gastrointestinal tract (GIT) conditions. Alongside GIT survival, natural bacterial decay was monitored for each isolate and control strain by performing viability assessments on both initial and final suspensions. This approach allowed spontaneous cell death to be accounted for throughout the assay period, ensuring that any reduction in viability could be accurately attributed to the specific stresses imposed by the GIT simulation. To evaluate the overall impact of simulated GIT conditions on bacterial viability, the change in viability between initial and final suspensions was analyzed for each strain using Welch’s two-sample t-test with Benjamini–Hochberg (BH) correction. Results are plotted in Figure 4.

Out of the 10 isolates tested, only three remained viable throughout all simulated gastrointestinal stages: *L. mesenteroides* (A1.64.2021 and A2.36.2021) and *E. hirae* (A1.8.2022) (Figure 4). The three surviving strains showed the most critical drop in cell viability during the Stomach 2 phase, where the pH decreased to 2.0. Statistical differences between the control strain and A1.64.2021 were found (*p* < 0.001), whereas no significant differences were found between the remaining isolates vs. the control strain. Furthermore, the lowest viability counts were recorded during the Intestine 1 and 2 stages, with significant differences between the control and strains (*p* < 0.05), likely due to the combined stress of pH 6.5, bile salts (3 g/L), and pancreatin (1 g/L) as well as recovery from the previous stage.The control strain exhibited statistical differences when comparing the initial and final suspensions, indicating bacterial decay. However, the viability drop was only of 0.54 log10 CFU, from an initial count of 7.63 ± 0.23 log10 CFU to 7.09 ± 0.15 log10 CFU at the final suspension (padj < 0.001). In contrast, none of the tested food isolates suffered population decay. Instead, all three isolates exhibiting minor but statistically significant increases in viable cell counts when comparing initial and final suspensions.

## 4. Discussion

This study was designed around two complementary objectives: rediscovering and preserving microbial heritage and subsequently unlocking its functional valorization. First, a representative collection of the culturable autochthonous microbiota associated with *Azeitão* and *Nisa* PDO cheeses produced during 2021 and 2022 was established to safeguard the indigenous bacterial diversity that underpins the authenticity, typicity, and terroir of these traditional products. These microorganisms constitute an essential component of artisanal cheesemaking, contributing not only to flavor and texture development but also to product safety and regional identity.

**Figure 5:**
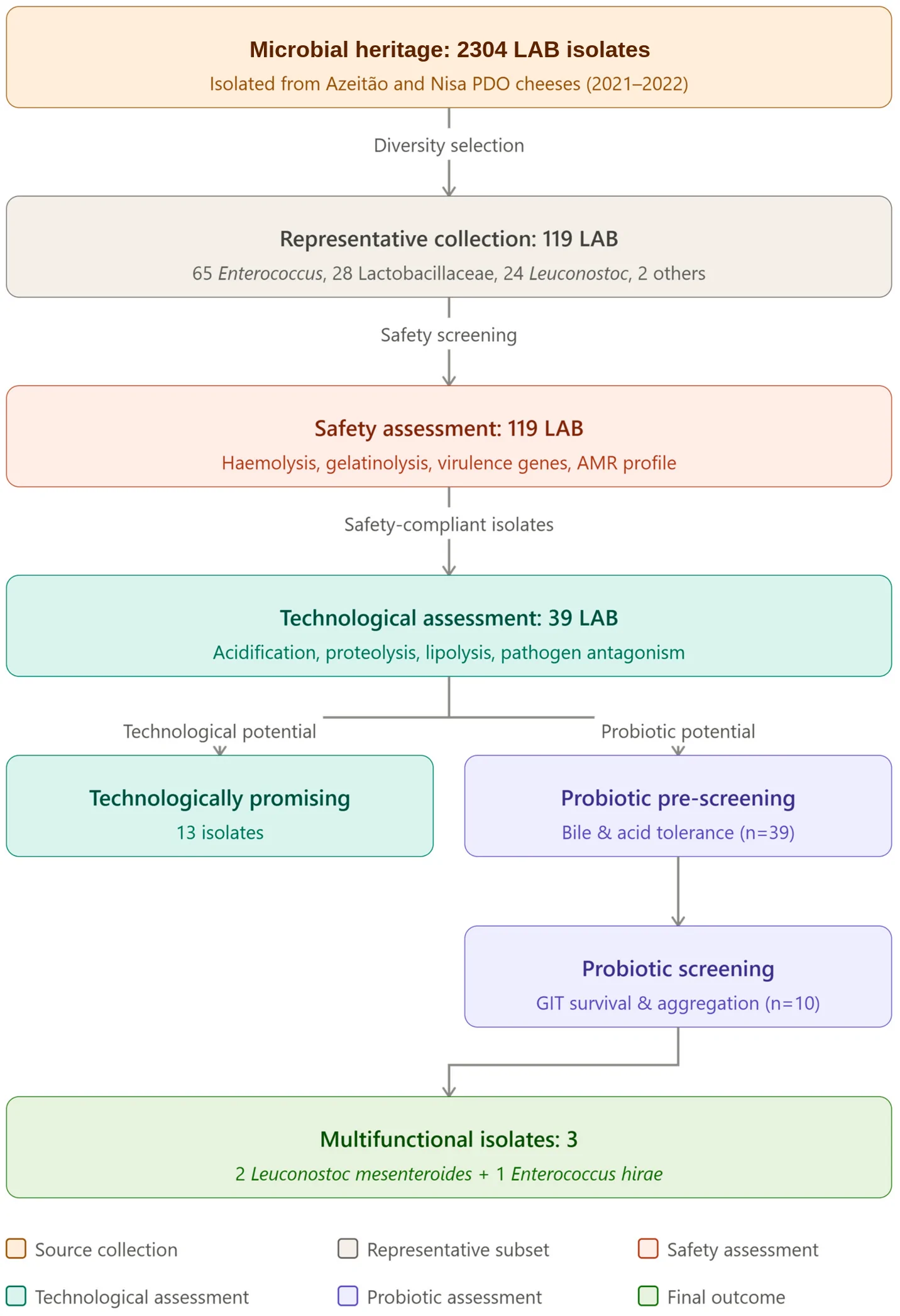
Polyphasic approach for the preservation and study of lactic acid bacteria present in PDO cheeses from Azeitão and Nisa produced in 2021 and 2022.

Building upon this microbial heritage collection, representative lactic acid bacteria (LAB) were subsequently subjected to a polyphasic characterization to identify strains with potential technological and probiotic value. Because PDO regulations prohibit the addition of exogenous microbial cultures during production, the preservation and characterization of indigenous LAB offer an opportunity to valorize these microbial resources beyond their original ecosystem. Selected strains may find future applications as starter, adjunct, protective, or probiotic cultures in non-PDO dairy products and other fermented foods, while preserving the unique microbial signatures associated with traditional cheesemaking. Given the intended food applications, only isolates meeting stringent safety criteria were advanced through successive stages of technological and probiotic evaluation. The findings from this integrated selection strategy are discussed in the following sections and summarized in Figure 5.

### 4.1. Lactic Acid Bacteria in PDO Cheeses from Azeitão and Nisa

Portuguese PDO cheeses rely on spontaneous fermentation by autochthonous microbiota, as the addition of external microorganisms is not permitted. This microbial complexity is responsible for the distinctive organoleptic properties of these products. While several PDO cheeses (e.g., *Serra da Estrela* [48,49], *Pico* [50,51], *Évora* [52] or *Serpa* [46,53,54]) have been extensively characterized, studies focusing on *Azeitão* and *Nisa* remain limited, particularly regarding the identification of strains with technological and probiotic potential. To our knowledge, this is the first study to perform a systematic screening of LAB from these regions integrating safety, technological and probiotic criteria, in PDO cheeses from two years of production.

Selected media (M17, MRS, and SBA) were effective for LAB recovery but showed limited specificity. In particular, M17 agar, conventionally used for lactococci or streptococci, predominantly yielded *Leuconostoc*, *Lactobacillaceae* sp., or *Enterococcus*. This lack of selectivity has been described by Ruggirello et al. [55,56] and is often attributed to the “Viable but Non-Culturable” (VBNC) state of lactococci during later stages of cheese ripening, alongside the proliferation of non-starter lactic acid bacteria (NSLAB), such as lactobacilli and enterococci. Similar observations regarding other Portuguese PDO cheeses by Kongo et al. (2007) [57], Domingos-Lopes et al. (2017) [23], Gonçalves et al. (2018) [53] and Salamandane et al. (2024) [58] support this interpretation and are consistent with our sampling of fully ripened cheeses, where *Lactococcus* strains were not detected despite high counts in M17 agar.

MRS medium supported the growth of multiple LAB genera beyond lactobacilli, including *Leuconostoc*, *Pediococcus*, and *Enterococcus*, confirming its broad selectivity. In contrast, SBA, designed for enterococci, yielded the lowest counts, particularly in 2022. Nevertheless, the frequent isolation of *Enterococcus* spp. is expected in raw milk cheeses, reflecting both environmental contamination and their ecological role as part of the cheese microbiota introduced during collection or manufacture. These bacteria are known to contribute to proteolysis, lipolysis, and antimicrobial activity, highlighting their dual technological and safety relevance (reviewed in Terzić-Vidojević et al. (2021) [59] and Serrano et al. 2024) [14]).

Overall, bacterial counts differed both between producers and production years, consistent with a highly dynamic microbiota that varies across producers and over time; as the present factorial design did not include producer as an independent statistical factor, this pattern is interpreted descriptively rather than as confirmatory evidence of a producer effect. While MRS counts remained relatively stable, variability observed in M17 and SBA suggests temporal and environmental influences on specific bacterial groups. These findings highlight the importance of combining culture-dependent approaches with functional screening to uncover the technological and probiotic potential of underexplored PDO cheese microbiota.

### 4.2. Safety Evaluation

Safety assessment was applied as an exclusion criterion in the selection of LAB isolates for technological and probiotic applications. Given the prevalence of *Enterococcus* spp. (*n* = 65) in the group of isolates, a genus lacking QPS status and requiring a case-by-case evaluation, screening focused on relevant virulence traits and antimicrobial resistance profiles combining phenotypic and genotypic approaches.

The absence of β-hemolytic activity across all tested isolates represents a favorable outcome, as hemolysin production is a relevant virulence trait in enterococci. Gelatinolytic activity was detected in a limited number of isolates, consistent with previous reports in cheese-derived enterococci [11,22]. At the genotypic level, *gel*E was the most frequently detected virulence determinant, in agreement with Gajewska et al. (2023) [60]; however, its presence did not always translate into a gelatinolytic phenotype. Similar discrepances were observed for *cyl*A, supporting reports in food isolates [22], likely reflecting gene silencing, regulatory variation, or truncated open reading frames, and reinforce the importance of combining both screening levels when assessing safety. The co-occurrence of *esp* and asa with *gel*E mirrors combinations previously described in cheese-associated *E. durans* strains [61], suggesting a shared genetic background in dairy-niche enterococci. Notably, 29 isolates harbored none of the virulence determinants screened, representing promising candidates for further technological and probiotic characterization.

Antimicrobial resistance profiling revealed distinct patterns between *Enterococcus* and non-enterococcal LAB. In enterococci, resistance to tetracycline and teicoplanin was relatively frequent, mirroring the findings of Gołaś-Prądzyńska et al. (2022) [62] in *E. faecalis* and *E. faecium* as well as the work performed by Bastião Rocha et al. (2022) [11]. In enterococci, tetracycline resistance is typically mediated by *tet* genes such as *tet*M, often associated with the Tn916/Tn1545 family of conjugative transposons [43], a mobility context that raises concern about horizontal transfer within the dairy environment and the wider food chain. In contrast, vancomycin resistance was rare. This contrasts with Salamandane et al. (2023) [63], who reported high vancomycin resistance rates in *E. faecalis*, and aligns more closely with the profile described by Bastião Rocha et al. (2022) [11]. Because teicoplanin and vancomycin remain critical last-line treatments for multidrug-resistant *E. faecium* infections [64], acquired glycopeptide resistance, even when limited to teicoplanin, warrants caution in foods. Regarding aminoglycosides, low-level resistance to streptomycin and gentamicin is reported as intrinsic to enterococci; however, the high screening concentrations used here indicate that the resistant profiles observed may reflect acquired mechanisms. However, phenotypic susceptibility testing alone cannot establish whether these determinants are transferable; further molecular and genomic characterization (e.g., screening for mobile genetic elements) would be required before any resistance mechanism is classified as acquired or potentially transmissible, consistent with recent recommendations for enterococcal isolates from fermented foods [65]. Reassuringly, no isolate displayed resistance to gentamicin at the high-level threshold, nor to linezolid, the latter being consistent with the largely clinical distribution of linezolid resistance reported in the literature [66]. In contrast to the *Enterococcus* collection, the non-enterococcal LAB isolates (*n* = 54) presented a resistance profile dominated by intrinsic, non-transferable mechanisms. Intrinsic vancomycin resistance is well documented in this family and arises from a structural modification of the cell wall, in which peptidoglycan precursors terminate in D-alanyl-D-lactate rather than D-alanyl-D-alanine, preventing glycopeptide binding to its target [46]. Consistently, most isolates were vancomycin-resistant; the few susceptible exceptions corresponded to *Lactiplantibacillus plantarum* strains and selected *Leuconostoc* isolates; this could be a strain-specific event. The high resistance frequencies observed for the aminoglycosides kanamycin, streptomycin, and gentamicin are likewise considered intrinsic traits, attributed to the absence of a cytochrome-mediated electron transport system that limits drug uptake (reviewed in Refaat et al. (2025) [67]). Because these mechanisms are chromosomally encoded and non-transmissible, they do not represent a biosafety concern and do not compromise the suitability of these isolates as technological or probiotic candidates. Resistance to ampicillin, chloramphenicol, and tetracycline in non-enterococcal LAB is, by contrast, typically acquired, requiring a careful analysis given the potential for horizontal transfer to opportunistic pathogens within the food matrix or the consumer’s gastrointestinal tract [68]. Non-enterococcal LAB were classified according to the breakpoints proposed by Touret et al. (2018) [26]. This precautionary choice may overestimate the phenotypically classified as resistant isolates among non-enterococcal LAB, ensuring that any isolate tested for technological and probiotic evaluation has cleared the strictest available safety evaluation. For the remaining resistances, the profiles were highly favorable: resistance to chloramphenicol, erythromycin, and clindamycin was essentially absent, and tetracycline resistance was restricted to a small number of isolates consequently excluded. This profile diverges from that reported by Nalepa and Markiewicz (2022) [69] in LAB from traditional Polish cheeses, where resistance rates reached 45.6% for tetracycline, 31.6% for chloramphenicol, and 22.8% for erythromycin, but aligns closely with observations on LAB from the Portuguese *Serpa* PDO cheese, where strains were fully or moderately susceptible to penicillin G, chloramphenicol, erythromycin, tetracycline, ampicillin, gentamicin, and clindamycin [46]. Together, the non-enterococcal LAB collection displayed the resistance profile expected from autochthonous dairy LAB: the extensive resistance to aminoglycosides described in literature, combined with lower percentages of resistance to other antibiotics, supports the safety baseline of the retained isolates for downstream probiotic evaluation.

After applying all safety criteria, including absence of virulence traits and exclusion of isolates with pathogenic potential, the candidate pool was substantially reduced. Notably, all *Enterococcus* isolates from the *Nisa* region and A4 producers in 2021 were excluded, primarily due to tetracycline resistance or the presence of virulence genes. This highlights significant variability in safety profiles between producers and production environments within the same PDO designation.

Overall, the complete collection revealed a highly diverse microbial community displaying markedly different safety profiles. As components of the complex cheese ecosystem, these autochthonous microorganisms collectively contribute to the microbial heritage of Portuguese PDO cheeses, where ecological interactions among species help maintain the balance and stability of the fermentation process. However, this ecological role does not necessarily translate into suitability for industrial application as individual cultures. When considered as standalone candidates for food or probiotic use, rigorous safety assessment becomes essential to exclude isolates carrying undesirable phenotypic or genotypic traits. These findings therefore highlight the importance of comprehensive safety screening as a prerequisite for the selection and valorization of autochthonous LAB from traditional cheeses.

The combined evaluation of virulence-associated traits and antimicrobial resistance profiles further refined the collection, demonstrating that only a limited proportion of the autochthonous isolates fulfilled the stringent safety requirements for subsequent functional characterization. These findings emphasize that, although traditional PDO cheeses constitute rich reservoirs of microbial diversity, only a small subset of indigenous LAB combines an appropriate safety profile with desirable technological and probiotic attributes. This reinforces the importance of comprehensive, strain-level evaluation through a multi-step selection strategy, rather than relying on species- or region-based assumptions, when identifying autochthonous cultures suitable for industrial applications.

### 4.3. Technological Evaluation

The technological screening revealed that a substantial portion of the isolated bacteria displayed traits associated with the development of flavor, texture and aroma in cheeses. The secretion of extracellular proteinases and lipases by LAB drives the primary biochemical pathways responsible for flavor and texture development during cheese ripening. As auxotrophic microorganisms, LAB have complex amino acid requirements that exceed the free concentrations naturally available in milk. Consequently, their persistence relies on an active proteolytic system capable of degrading caseins into small peptides and free amino acids, which concurrently generates the primary aroma and flavor precursors in fermented dairy products [70]. Simultaneously, lipolysis facilitates the hydrolysis of milk fats, primarily triacylglycerols, into free fatty acids. These fatty acids and amino acids are subsequently catabolized into volatile secondary metabolites, including esters, aldehydes, alcohols, and ketones, that define the final sensory profile of ripened cheese [71]. When comparing these enzymatic capabilities to the literature, the proportion of proteolytic-positive *Lactiplantibacillus plantarum* isolates observed in this study was lower than that reported by Grujović et al. (2024) [72], who identified a high prevalence of proteolytic activity among *Lactiplantibacillus plantarum* and *Lacticaseibacillus paracasei* isolates. This discrepancy may reflect differences in the cheese matrix of origin, ripening stage at sampling, or strain-level variability. Conversely, the caseinolytic activity detected in the *E. faecalis* (A3.4.2021) isolate is consistent in prevalence with the observations of Câmara et al. (2019) [51], reinforcing the well-documented proteolytic contribution of enterococci in artisanal cheeses. A clear divergence emerged, however, regarding lipid metabolism: whereas Câmara et al. (2019) [51] reported an absence of lipolytic activity among their isolates and only four strains combining both proteolytic and lipolytic traits, the current collection contained a comparable number of dual-active isolates, four non-enterococcal LAB (A3.54.2021, A3.55.2021—*Lactobacillaceae* sp., A2.24.2021—*Lactiplantibacillus plantarum*) and one *E. faecalis* (A3.4.2021), positioning them as the strongest NSLAB candidates within this collection. Even so, since both activities are not detrimental when selecting NSLAB, the distribution of lipolytic activity across a broader fraction of our isolates may indicate adaptation to the specific lipid-rich environment of the sampled cheeses and could translate into a contribution to secondary aroma formation during ripening. Integrating these enzymatic traits with the thermal and salt-tolerance assays further shows the NSLAB profile of the collection. Growth at 10 °C, simulating maturation chamber conditions used in both regions (*Azeitão* 10–12 °C; *Nisa* 8–10 °C in the first phase and 12–14 °C in the second), is a requirement for NSLAB, since isolates unable to multiply at this temperature are unlikely to persist or contribute meaningfully during ripening. Thus, isolate A3.55.2021 was effectively excluded from the NSLAB candidate pool despite its dual enzymatic activity. Regarding salt tolerance, some isolates, specifically those recovered from A2 in 2021, showed reduced survival at a higher NaCl concentration (5%). This NaCl concentration was used to test the maximum adaptability of cheese isolates to salt concentrations and does not reflect the actual salt concentrations found in *Azeitão* and *Nisa* cheeses (between 1 and 2.5%). Thus, this lack of adaptation was not considered as a factor for direct exclusion; however, it could reflect factory-specific environmental conditions, differences in brining practices, or shifts in the resident microbiota between sampling periods. Taken together, the combined enzymatic, thermal, and osmotic profiles converge on a small subset of isolates, particularly *Leuconostoc* sp. A2.12.2021, *Lactobacillaceae* sp. A3.54.2021, *Lactiplantibacillus plantarum* A2.24.2021, and *E. faecalis* A3.4.2021, as the most promising candidates for further evaluation as adjunct NSLAB cultures.

Another aspect evaluated in this work was a screening for inhibitory activity against pathogenic and spoilage bacteria, a trait that attests to the biopreservative potential of LAB and is increasingly valued in the selection of adjunct cultures for dairy production and safety. In the present collection, 10 isolates (seven non-enterococcal LAB—A1.50.2021, A2.6.2022, A2.30.2022, N9.3.2021, N9.1.2022, N9.49.2022 (all *Lactobacillaceae* sp.), A4.8.2022 (*L. mesenteroides*), and two *Enterococcus*—A1.8.2022 (*Enterococcus* sp.) and A1.55.2022 (*E. faecalis*)) displayed inhibitory activity against all indicator strains tested, suggesting a broad-spectrum protective capacity. This trait is particularly relevant for PDO Portuguese traditional cheeses, where the use of raw milk relies on the autochthonous microbiota of the milk and producer environment to exert selective pressure against potentially pathogenic or deteriorative microorganisms. Previous studies have extensively explored the competitive potential of cheese-isolated LAB against various indicator strains. De Sant’Anna et al. (2017) [73] demonstrated that LAB isolates from Brazilian artisanal cheeses, predominantly former *Lactobacillus* genera and *Pediococcus*, exerted antimicrobial activity against both *E. faecalis* and *E. coli*. Similarly, Dias et al. (2019) [74] reported that *E. faecalis* M173, isolated from sheep milk, inhibited a broad range of indicators, including *E. coli*, *L. monocytogenes*, and *P. aeruginosa*. These observations are consistent with the present study, in which the *E. faecalis* (A1.55.2022) and *E. hirae* (A1.8.2022) isolates likewise displayed broad-spectrum inhibition. Câmara et al. (2019) [51], working with LAB from *Pico* PDO cheese, observed inhibitory activity against *E. coli* and *L. monocytogenes* across their entire collection of *Enterococcus*, former *Lactobacillus*, and *Leuconostoc pseudomesenteroides* isolates. In the current work, inhibition of *E. coli* and *L. monocytogenes* was less uniformly distributed across the collection; this divergence regarding *L. monocytogenes* may, at least in part, reflect the use of different indicator strains (CECT 935 in the present study *versus* ATCC 7644 in Câmara et al. (2019) [51]) and inter-strain variability in *Listeria* susceptibility to LAB-derived antimicrobials. Regarding *Pseudomonas*, Câmara et al. (2019) [51] reported smaller inhibition zones for *P. aeruginosa* compared with other indicators; in the present study, *P. fluorescens* was selected instead due to its primary role in dairy spoilage rather than its clinical relevance, and a similarly reduced inhibitory efficacy was observed relative to the remaining indicators. In contrast, *B. cereus* emerged as the second-most inhibited indicator in the non-enterococcal LAB collection, which differs from the findings of Araújo-Rodrigues et al. (2021) [54], where only two *L. plantarum* isolates were active against this pathogen. This higher prevalence of anti-*B. cereus* activity may reflect the specific composition of the *Azeitão* and *Nisa* microbiota and reinforces the relevance of these isolates for the control of spoilage and pathogenic bacteria in dairy environments.

A notable feature of the present results is that overall antimicrobial activity was lower against *L. monocytogenes*, in the case of non-enterococcal LAB, than against the Gram-negative indicators tested. This pattern is atypical given that LAB are generally considered more effective against Gram-positive bacteria, due to the absence of an outer membrane barrier [75,76]. Although the screening performed here did not include characterization of the inhibitory compounds, plausible explanations include strain-specific resistance of *L. monocytogenes* CECT 935 or differences in the nature and spectrum of the antimicrobial metabolites produced by the isolates under the assayed conditions used [77,78].

A further point of interest concerns intra-genus inhibition: while several non-enterococcal LAB inhibited the *E. faecalis* indicator, only five enterococcal isolates displayed this capacity, showing a limited capacity of cheese enterococci to inhibit other *Enterococcus* species. This shows the importance of a dynamic microbial community within the cheeses and their implication in the safety of PDO cheeses. Intra-genus antagonism among enterococci has been reported in other contexts, including the work of Fujii et al. (2025) [79], in which clinical *E. faecalis* and *E. faecium* strains selectively inhibited other enterococci while showing no activity against *E. coli*, *Acinetobacter baumannii*, *Staphylococcus epidermidis*, or *S. aureus*. The screening approach used in the present study does not allow conclusions regarding the underlying mechanisms, but the observation that intra-genus inhibition was comparatively rare among the cheese enterococci is noteworthy and may merit dedicated investigation in future work. Overall, the cheese isolates from *Azeitão* and *Nisa* display an inhibitory profile of interest for cheese safety, particularly against Gram-negative targets and *B. cereus*, and warrant further investigation into the underlying antimicrobial mechanisms. Moreover, this fact also attests to the importance and influence of these bacteria in PDO cheeses, since by using their inhibitory effects during cheese production, they confer increased control of pathogenic or deteriorative bacteria without disturbing the natural autochthonous microbiota already in place.

Differences between producers and production years were also apparent in the inhibition profiles. Within the *Enterococcus* collection, broad-spectrum inhibition was restricted to A1 isolates from 2022, whereas in the N9 isolates, a high proportion of 2021 strains and a smaller fraction of 2022 strains showed inhibitory activity. These producer- and year-dependent patterns reinforce the notion that the specific environment of each cheese-making facility plays a determinant role in shaping the functional potential of its autochthonous microbiota.

Overall, the technological evaluation identified a subset of isolates combining enzymatic activity, environmental resilience, acidification capacity, and antimicrobial potential, reinforcing the value of integrating multiple functional criteria when selecting autochthonous LAB as adjunct cultures; the specific isolates showing the strongest combined profiles are highlighted above (Section 4.3).

### 4.4. Probiotic Evaluation

The characterization of multifunctional LAB does not end with the assessment of their technological performance. To be considered suitable probiotic candidates, strains must not only withstand the manufacturing process and remain viable throughout product storage but also survive gastrointestinal transit in sufficient numbers to exert beneficial effects on the host. Consequently, probiotic selection requires the evaluation of traits associated with persistence under gastrointestinal conditions, as well as the ability to interact with the intestinal environment [80].

In the present study, probiotic potential was initially assessed through a series of in vitro screening assays. Compared with the technological evaluation, only a limited number of isolates fulfilled the criteria required to be considered promising probiotic candidates, highlighting the greater stringency of probiotic selection. Two *L. mesenteroides* (A1.64.2021, A2.36.2021) and one *E. hirae* (A1.8.2022) were the only isolates that showed resilience within the simulated gastrointestinal tract environment. In contrast to previous studies, none of the *Lactobacillaceae* sp., strains in our collection survived the complete GIT simulation. However, our findings regarding *L. mesenteroides* align with those of Le & Yang (2019) [81], who characterized *L. mesenteroides* FB111 as a strain highly adapted to gastrointestinal conditions. Similarly to our observations for isolate A1.64.2021, these authors reported high initial viability (log_10_ 9 CFU/mL) during the mouth stage. While Le & Yang (2019) [81] observed a relatively stable decline through the gastric (log_10_ 8.8 to 7.9 CFU/mL) and intestinal (log_10_ 7.7 CFU/mL) phases, our collection exhibited a more pronounced reduction in viable counts when transitioning from the stomach to the intestine. This decline in our isolates suggests a higher sensitivity to the synergistic stress of bile salts and pancreatic enzymes, despite the initial resilience shown during the gastric phase. Strains of probiotic *E. hirae* have been isolated from goat and sheep milk [82]; nonetheless, when Portuguese traditional cheeses are concerned, usually the enterococci collections, although the more prevalent, are completely excluded during safety evaluation [23,51]. In the case of this collection, 10 enterococci were considered safe, and one showed adaptability towards bile, pH and gastrointestinal tract survival, being considered an interesting candidate to continue to explore regarding probiotic potential. Nonetheless, particular caution is warranted regarding this *Enterococcus hirae* isolate: the current phenotypic and limited genotypic safety screening is not sufficient to establish its suitability for probiotic use, and further molecular or genomic characterization of virulence factors and antimicrobial resistance determinants would be necessary before this isolate could be considered for any application. Regarding, aggregation capabilities, all except A1.8.2022 (which had moderate auto-aggregation) showed low auto-aggregation. As for co-aggregation with indicator bacteria, A1.8.2022 again exhibited interesting results since it showed moderate capability to aggregate with *E. coli*, *L. monocytogenes* and *S. enteritidis*. However, in the case of A1.64.2021, only moderate aggregation was observed with *E. coli*, and in the case of A2.36.2021, moderate aggregation was observed with *E. coli* and *S. enteritidis*.

Although probiotic screening yielded only a limited number of candidates, three autochthonous LAB isolates combined desirable technological and probiotic-related traits, highlighting their potential as multifunctional cultures for future dairy and probiotic applications. Their ability to tolerate industry-relevant and simulated gastrointestinal conditions, together with their antimicrobial activity, suggests that they could be explored as adjunct or protective cultures in non-PDO dairy products, contributing simultaneously to product quality, biopreservation, and consumer health.

Nevertheless, these findings represent only an initial step in probiotic characterization. In vitro studies are still required to evaluate additional functional attributes, including antioxidant activity, immunomodulatory effects, cholesterol assimilation, and adhesion to intestinal epithelial cell lines (e.g., *Caco-2*), together with whole-genome sequencing of the three candidate isolates to confirm the absence of transferable antimicrobial resistance determinants and mobile genetic elements, followed by well-designed in vivo studies to confirm their efficacy and safety. More importantly, these results demonstrate that preserving the microbial heritage of traditional PDO cheeses creates a valuable reservoir of indigenous microorganisms from which rare multifunctional strains can be identified and scientifically validated for future applications, while safeguarding the microbial biodiversity that defines the identity and terroir of these artisanal products.

## 5. Conclusions

This study established a comprehensive collection of 2304 autochthonous bacterial isolates from Azeitão and Nisa PDO cheeses produced over two consecutive years, creating a valuable repository of microbial resources that reflects the biological diversity underpinning these traditional products. The observed variability in the recovered microbiota between producers and production years highlights the dynamic nature of these artisanal ecosystems and reinforces the importance of preserving their indigenous microbial communities as an integral component of cheese terroir.

Through a systematic polyphasic characterization, representative lactic acid bacteria were evaluated for safety, technological performance, and probiotic-related traits, identifying 39 isolates with promising technological characteristics and three multifunctional candidates warranting further investigation. However, the greatest contribution of this work extends beyond the identification of strains with biotechnological potential. By documenting, preserving, and functionally characterizing these indigenous microorganisms, this study rediscovers and safeguards a hidden dimension of the microbial heritage of Portuguese PDO cheeses, providing a scientific foundation for its future valorization.

Pending further genomic safety assessment, validation in food matrices, and in vivo studies, these autochthonous LAB, and particularly the three multifunctional candidates identified here, should be regarded as promising components of Portugal’s gastronomic heritage and as candidates worth exploring for future dairy innovation. Preserving these microbial resources is essential to maintain the authenticity, biodiversity, and resilience of traditional cheesemaking systems, ensuring that the microbial signatures defining the unique identity of PDO cheeses are conserved for future generations. In this sense, this rediscovered terroir links microbial biodiversity with cultural heritage and future food innovation, revealing indigenous microorganisms as both witnesses of the past and resources for the future.

## Figures and Tables

**Table 1:** Genus- and species-level identification of the LAB isolates.

Identification	2021						2022					
	Producers						Producers					
	A1	A2	A3	A4	N9	N10	A1	A2	A3	A4	N9	N10
*Lactobacillaceae* sp. (*n* = 24)	2		3	1	3		2	2	2	2	3	4
*Lactiplantibacillus plantarum* (*n* = 4)		1								1	2	
*Lacticaseibacillus paracasei* (*n* = 1)				1								
*Leuconostoc* sp. (*n* = 17)		2	4	2	1	2			3	1		2
*Leuconostoc mesenteroides* (*n* = 7)	1	3					1			2		
*Pediococcus* sp. (*n* = 1)									1			
*Enterococcus* sp. (*n* = 5)	2			1	1		1					
*E. faecalis* (*n* = 48)	4	1	6	5	1	7	3	5	5	6	3	2
*E. faecium* (*n* = 2)		2										
*E. durans* (*n* = 9)	1	1	1		2			1		3		
*E. hirae* (*n* = 1)							1					

## Data Availability

The original contributions presented in this study are included in the article/Supplementary Materials. Further inquiries can be directed to the corresponding author.

## Supplementary Materials

The following supporting information can be downloaded at: https://www.mdpi.com/article/10.3390/foods15183322/s1, Table S1: Bacterial enumeration of PDO cheeses produced in *Azeitão* and *Nisa*; Table S2: Safety assessment of cheese Enterococcus isolates; Table S3: Technological assessment of LAB, cheese factory simulated conditions, as well as proteolytic and lipolytic activities; Table S4: Technological assessment of enterococci, cheese factory simulated conditions, as well as proteolytic and lipolytic activities; Table S5: Survival screening and inhibition potential of non-enterococcal lactic acid bacteria (LAB) against pathogenic and deteriorative bacteria; Table S6: Survival screening and inhibition potential of *Enterococcus* against pathogenic and deteriorative bacteria; Table S7: Post hoc pairwise comparisons of aggregation percentages across incubation periods.
