# Supplementary material for: Terroir Rediscovered: A Polyphasic Approach to Autochthonous Lactic Acid Bacteria as Functional and Microbial Heritage Resources in Portuguese PDO Cheeses
Source: Foods. 2026 Sep 19;15(18):3322. doi: 10.3390/foods15183322 (PMC13606075; doi:10.3390/foods15183322)
Supplement: Supplementary file 1 [file foods-15-03322-s001.zip › foods-4502793-supplementary.pdf]

Supplementary Table S1 - Bacterial enumeration of PDO cheeses produced in *Azeitão* and *Nisa*

Media	Year	Region	Factory	Counts	Log10 (CFU/g)
M17	2021	*Azeitao*	A1	4930000,00	6,69284692
M17	2021	*Azeitao*	A2	1226666,67	6,08872656
M17	2021	*Azeitao*	A3	7300000,00	6,86332286
M17	2021	*Azeitao*	A4	14010000,00	7,14643814
M17	2021	*Nisa*	N9	1260000,00	6,10037055
M17	2021	*Nisa*	N10	188666,67	5,27569518
M17	2022	*Azeitao*	A1	11200000,00	7,04921802
M17	2022	*Azeitao*	A2	200000,00	5,30103
M17	2022	*Azeitao*	A3	10100000,00	7,00432137
M17	2022	*Azeitao*	A4	4000000,00	6,60205999
M17	2022	*Nisa*	N9	5466666,67	6,73772259
M17	2022	*Nisa*	N10	11200000,00	7,04921802
MRS	2021	*Azeitao*	A1	5300000,00	6,72427587
MRS	2021	*Azeitao*	A2	9300000,00	6,96848295
MRS	2021	*Azeitao*	A3	4950000,00	6,6946052
MRS	2021	*Azeitao*	A4	5860000,00	6,76789762
MRS	2021	*Nisa*	N9	798000,00	5,90200289
MRS	2021	*Nisa*	N10	3620000,00	6,55870857
MRS	2022	*Azeitao*	A1	15500000,00	7,1903317
MRS	2022	*Azeitao*	A2	14850000,00	7,17172645
MRS	2022	*Azeitao*	A3	17200000,00	7,23552845
MRS	2022	*Azeitao*	A4	12300000,00	7,08990511
MRS	2022	*Nisa*	N9	13500000,00	7,13033377
MRS	2022	*Nisa*	N10	43300000,00	7,6364879
SBA	2021	*Azeitao*	A1	1380000	6,13987909
SBA	2021	*Azeitao*	A2	9700000	6,98677173
SBA	2021	*Azeitao*	A3	1200000	6,07918125
SBA	2021	*Azeitao*	A4	7300000	6,86332286
SBA	2021	*Nisa*	N9	2700000	6,43136376
SBA	2021	*Nisa*	N10	9700000	6,98677173
SBA	2022	*Azeitao*	A1	20000	4,30103
SBA	2022	*Azeitao*	A2	150000	5,17609126
SBA	2022	*Azeitao*	A3	130000	5,11394335
SBA	2022	*Azeitao*	A4	140000	5,14612804
SBA	2022	*Nisa*	N9	8800	3,94448267
SBA	2022	*Nisa*	N10	7000000	6,84509804

Supplementary Table S2 - Safety assessment of cheese *Enterococcus* isolates. Resistance to antibiotics was measuered in milimiters and determined using CLSI (2020) breakpoints, for isolates that showed resistance to vancomycin and/or linezolid E-test validation was also performed. Susceptibility is highlighted in green; Intermediate resistance in yellow; Resistance in red.

Species ID	Isolates	Hemolytic activity	Gelatinolitic activity	Virulence genes	Amp (10 µg)	CIP (5 µg)	C (30 µg)	E (15 µg)	S (300 µg)	CN (120 µg)	LEV (5 µg)	LZD (30 µg)	QD (15 µg)	TEC (30 µg)	TE (30 µg)	VA (30 µg)	E-test VAN	E-test LZD
Enterococcus faecalis	A1.2 2021	*α*	Negative	-	25	17	20	19	10	15	20	22	12	10	11	14	I	
Enterococcus faecalis	A1.24 2021	*α*	Negative	*gel*E	22	17	20	17	9	18	18	22	20	15	22	14	S	
Enterococcus durans	A1.45 2021	*α*	Negative	-	30	20	20	20	13	16	21	25	15	16	21	15		
Enterococcus faecalis	A1.59. 2021	*α*	Negative	-	33	22	22	21	14	15	20	25	15	15	0	15		
Enterococcus faecium	A2.3 2021	*λ*	Negative	-	30	12	21	15	15	20	12	21	15	12	16	16		
Enterococcus durans	A2.17 2021	*λ*	Negative	-	16	20	20	13	15	20	25	17	16	15	15	15		S
Enterococcus faecium	A2.32 2021	*λ*	Negative	-	28	16	25	15	20	17	16	22	19	13	20	15		
Enterococcus faecalis	A2.58 2021	*λ*	Negative	-	30	25	22	30	25	22	25	21	22	19	25	17		
Enterococcus faecalis	A3.4 2021	*λ*	Negative	-	25	16	16	20	12	13	17	21	11	13	19	13	S	
Enterococcus durans	A3.9 2021	*λ*	Negative	-	18	25	15	15	10	12	20	25	15	14	10	15		
Enterococcus faecalis	A3.16 2021	*λ*	Negative	*gel*E	25	14	20	15	11	11	15	21	11	12	10	14	S	
Enterococcus faecalis	A3.19 2021	*λ*	Negative	*gel*E	30	17	22	15	11	19	21	22	16	10	0	14	I	

Enterococcus faecalis	A3.26 2021	λ	Negative	*gel*E	20	15	9	11	0	14	16	21	17	11	0	14	S	
Enterococcus faecalis	A3.39 2021	λ	Negative	*gel*E	20	16	16	15	10	13	15	21	14	14	0	14	I	
Enterococcus faecalis	A3.45 2021	λ	Negative	*gel*E	20	15	20	18	11	12	15	25	17	15	10	14	S	
Enterococcus faecalis	A4.7 2021	λ	Positive	*gel*E	22	20	0	0	0	15	22	21	11	15	0	14	S	
Enterococcus faecalis	A4.11 2021	α	Negative	*gel*E	22	16	19	16	0	12	15	21	15	14	10	14	S	
Enterococcus faecalis	A4.21 2021	α	Positive	*gel*E	21	15	19	15	11	13	15	22	10	14	19	14	S	
Enterococcus faecalis	A4.26 2021	α	Negative	*gel*E	20	15	16	15	0	12	14	21	13	10	9	11	S	
Enterococcus faecalis	A4.41 2021	α	Negative	*gel*E	20	15	19	19	0	11	16	21	14	11	9	12	R	
Enterococcus durans	N9.21 2021	α	Negative	-	25	20	>30	40	25	30	18	35	25	0	12	0	R	
Enterococcus durans	N9.46 2021	α	Negative	-	22	25	30	40	30	40	20	40	20	10	0	0	R	
Enterococcus faecalis	N9.58 2021	α	Negative	-	25	20	>30	35	25	30	20	30	25	8	10	13	R	
Enterococcus faecalis	N10.1 2021	λ	Negative	-	22	15	10	0	0	11	15	25	11	14	0	11	S	
Enterococcus faecalis	N10.21 2021	λ	Negative	*gel*E	20	20	0	8	0	12	15	21	10	13	0	12	S	
Enterococcus faecalis	N10.45 2021	λ	Negative	*gel*E	20	16	20	15	12	10	16	20	12	12	9	12	S	S
Enterococcus faecalis	N10.47 2021	λ	Negative	-	25	18	15	19	10	10	15	19	15	15	10	14	S	S

Enterococcus faecalis	N10.49 2021	α	Positive	*gel*E	21	0	19	15	0	12	15	21	10	10	9	12	S	
Enterococcus faecalis	A1.14 2022	α	Positive	*gel*E	20	17	20	22	13	14	18	21	11	10	20	13	S	
Enterococcus sp.	A1.54 2022	λ	Negative	-	22	21	20	21	10	18	16	21	11	10	0	15		
Enterococcus durans	A2.1 2022	λ	Negative	-	21	16	18	15	11	12	17	21	12	11	20	12	S	
Enterococcus faecalis	A2.23 2022	λ	Negative	*gel*E	25	15	20	16	11	12	15	25	11	11	10	11	S	
Enterococcus faecalis	A2.34 2022	λ	Positive	-	20	17	20	18	13	14	16	25	14	15	19	17		
Enterococcus faecalis	A2.38 2022	λ	Negative	*gel*E	22	18	20	15	20	12	15	22	10	12	0	10	S	
Enterococcus durans	A4.7.2021	λ	Negative	-	22	20	0	0	0	15	22	21	11	15	0	14	S	
Enterococcus faecalis	A3.14 2022	λ	Negative	-	21	18	20	15	10	15	20	22	10	15	0	15		
Enterococcus faecalis	A3.47 2022	λ	Negative	-	25	16	18	20	11	14	20	21	14	15	20	16		
Enterococcus faecalis	A3.49 2022	λ	Negative	-	20	15	20	17	11	10	15	21	11	11	0	11	I	
Enterococcus faecalis	A3.57 2022	λ	Negative	-	20	18	18	21	11	15	18	25	14	14	19	14	I	
Enterococcus faecalis	A4.5 2022	λ	Positive	*gel*E	19	19	10	0	0	13	17	21	10	11	0	11	S	
Enterococcus durans	A4.11 2022	λ	Positive	*gel*E, *cyl*A, *esp*	17	16	16	15	10	12	16	21	11	11	18	10	S	
Enterococcus faecalis	A4.12 2022	λ	Positive	*gel*E	21	18	15	12	0	11	20	19	11	12	14	11	S	S
Enterococcus durans	A4.20 2022	α	Negative	-	22	15	15	20	13	13	20	21	14	14	15	15		

Enterococcus faecalis	A4.30 2022	λ	Positive	*gel*E	22	16	16	18	11	10	15	21	11	11	9	10	I	
Enterococcus faecalis	A4.43 2022	α	Negative	*gel*E	21	17	20	14	10	14	17	25	12	12	9	11	I	
Enterococcus faecalis	A4.54 2022	α	Negative	*gel*E	25	19	21	20	10	12	19	21	12	14	9	14	I	
Enterococcus faecalis	A4.59 2022	α	Negative	*gel*E	25	17	16	16	10	12	18	19	13	12	15	11	I	S
Enterococcus faecalis	N9.1 2022	λ	Positive	*gel*E	20	17	10	0	12	15	16	22	10	15	8	15		
Enterococcus faecalis	N9.23 2022	λ	Positive	*gel*E, *agg*	26	20	10	12	14	15	20	25	11	19	10	15		
Enterococcus faecalis	N9.58 2022	α	Positive	*gel*E, *agg*	30	20	0	11	11	15	20	25	10	15	0	16		
Enterococcus faecalis	N10.18 2022	λ	Negative	*gel*E, *cyl*A	22	19	18	15	12	15	18	25	10	18	12	14	S	
Enterococcus faecalis	N10.55 2022	λ	Negative	*gel*E	25	22	22	25	12	15	20	27	20	15	10	15		
Enterococcus faecalis	A3.38 2022	λ	Negative	-	25	22	20	15	10	14	20	21	12	14	0	14	R	
Enterococcus sp.	A4.18. 2021	α	Negative	*gel*E, *esp*	13	19	39	34	0	15	19	22	20	16	37	0	R	
Enterococcus faecalis	N10.58 .2021	λ	Negative	*gel*E, *esp*	24	32	0	0	44	18	31	26	40	16	27	30		
Enterococcus faecalis	N10.17 .2021	α	Positive	*gel*E, *esp*	28	21	47	23	0	16	23	26	12	16	22	28		
Enterococcus faecalis	A1.11. 2021	λ	Negative	-	23	20	30	37	21	28	26	30	23	20	31	0	R	
Enterococcus sp.	A1.13. 2021	λ	Negative	-	25	24	37	33	24	25	24	40	26	27	36	26		
Enterococcus sp.	A1.63. 2021	λ	Negative	*cyl*A	21	24	28	30	20	25	25	30	22	20	31	22		

Enterococcus hirae	A1.8.2 022	λ	Negative	-	24	30	30	30	25	30	30	35	25	22	32	22	
Enterococcus faecalis	A1.51. 2022	λ	Negative	*gel*E, *agg*	22	25	32	30	15	18	25	32	13	19	31	19	
Enterococcus faecalis	A1.55. 2022	λ	Negative	-	23	24	27	27	14	19	24	26	13	18	28	18	
Enterococcus faecalis	A2.36. 2022	λ	Negative	*gel*E	24	23	29	22	12	24	17	27	10	13	29	13	R
Enterococcus faecalis	A2.40. 2022	λ	Positive	*gel*E	26	24	30	24	15	13	25	30	10	20	33	19	
Enterococcus sp.	N9.26. 2021	α	Positive	-	24	30	35	33	25	31	29	40	23	27	13	25	

Supplementary Table S3 - Technological assessment of LAB, cheese factory simulated conditions as well as proteolytic and lipolytic activities.

Species ID	Isolates	Temperature				NaCl			Acidification			Lipolitic activity	Proteolitic activity
		4	10	20	37	1%	2.5%	5%	2h	6h	24h		
Lactobacillus sp	A1.50.2021	-	-	+	+	+++	+++	+++	7	6	4	-	-
Lactobacillus sp	A2.06.2022	+	-	+	+	+++	++	++	6	6	6	-	-
Lactobacillus sp	A2.30.2022	+	+	+	+	+	+	+	7	6	6	-	-
Lactobacillus sp	A3.54.2021	-	+	+	+	++	++	+	7	6	5	+	+
Lactobacillus sp	A3.55.2021	+	-	+	+	++	++	+	7	6	5	+	+
Lactobacillus sp	A3.37.2022	-	-	+	+	+++	+++	+++	7	6	6	+	-
Lactobacillus sp	A3.61.2022	-	-	+	+	+++	+++	++	7	6	6	+	-
Leuconostoc mesenteroides	A4.8.2022	+	+	+	+	++	+++	-	7	6	6	+	-

Leuconostoc mesenteroides	A4.41.2022	-	-	+	+	++	+++	+++	7	6	4	+	-
Lactobacillus sp	N9.3.2021	+	-	+	+	+++	+++	+++	7	6	6	+	-
Lactobacillus sp	N9.1.2022	-		+	+	+++	+++	++	7	6	5	-	-
Lactobacillus sp	N9.29.2022	-	+	+	+	+++	+++	++	7	6	5	-	-
Lactobacillus sp	N9.49.2022	-	-	+	+	+++	+++	+	6	6	4	-	-
Leuconostoc mesenteroides	A1.64.2021	-	+	+	+	+++	+++	+++	6	6	5	+	-
Leuconostoc	A2.12.2021	+	+	+	-	+++	++	-	6	6	5	-	+
Lactobacillus plantarum	A2.24.2021	+	+	+		+++	+++	+++	6	6	5	+	+
Leuconostoc	A2.29.2021	+	+	+	-	++	++	-	6	6	5	-	-
Leuconostoc mesenteroides	A2.36.2021	+	+	+	+	+++	+++	-	6	6	5	+	-
Pediococcus sp	A3.3.2022	+	+	+	+	++	++	++	6	6	5	+	-
Leuconostoc	A3.61.2022	+	+	+	+	+++	+++	+++	6	6	5	+	-
Leuconostoc	A4.5.2021	+	+	+	+	+++	+++	+++	6	6	5	-	-
Lactobacillus sp	A4.46.2021	+	+	+	+	+++	+++	+++	6	6	5	+	-
Lactobacillus plantarum	A4.24.2022	+	+	+	+	+	+	+	6	6	5	+	-
Lactobacillus sp	A4.38.2022	+	+	+	+	++	++	++	6	6	5	+	-
Lactobacillus plantarum	N9.10.2022	+	+	+	+	+++	+++	-	6	6	4	-	-
Lactobacillus plantarum	N9.58.2022	+	+	+	+	+++	+++	+	6	6	4	-	-
Leuconostoc	N10.59.2021	+	+	+	+	+++	+++	+	6	6	5	+	-

Leuconostoc	N10.6.2022	+	+	+	+	+++	++	++	6	6	5	-	-
Lactobacillus sp	N10.15.2022	+	+	+	+	+++	++	++	6	6	5	-	-

Supplementary Table S4 - Technological assessment of enterococci, cheese factory simulated conditions as well as proteolytic and lipolytic activities. Positive results in temperature and NaCl were considered when bacterial multiplication was evident; In the case of lipolytic activity, blue or transparent halos were considerate positive results; In the case of proteolytic activity, the appearance of halos was considerate a positive result.

Species ID	Isolates	Temperature (°C)				NaCl			Milk acidification (pH±0.5)			Lipolytic activity	Proteolytic activity
		4	10	20	37	1%	2.5%	5%	2h	6h	24h		
E. durans	A1.45 2021	-	+	+	+	+++	+++	+++	6	6	5	-	-
E. faecium	A2.32 2021	+	+	+	+	+++	+++	+++	6	6	5	+	-
E. faecalis	A2.58 2021	+	+	+	+	+++	+++	+++	6	6	5	+	-
E. faecalis	A3.4 2021	-	+	+	+	+++	+++	+++	6	6	5	+	+
E. durans	A2.1 2022	+	+	+	+	+++	+++	+++	6	6	5	-	-
E. faecalis	A3.47 2022	+	+	+	+	+++	+++	+++	6	6	5	+	-
E. durans	A4.20 2022	+	+	+	+	+++	+++	+++	6	5	5	+	-
Enterococcus sp.	A1.13.2021	+	+	+	+	+++	+++	+++	6	6	5	+	-
Enterococcus sp.	A1.8.2022	+	+	+	+	+++	+++	+++	6	6	5	+	-
E. faecalis	A1.55.2022	+	+	+	+	+++	+++	+++	6	6	5	+	-

Supplementary Table S5 – Survival screening and inhibition potential of non-enterococcal lactic acid bacteria (LAB) against pathogenic and deteriorative bacteria. Positive results in bile salts and acidic pH were defined by evident bacterial multiplication. Inhibition zones against pathogenic and deteriorative bacteria were measured in millimetres.

Species ID	Isolates	Bile salts (0.5%)	pH=2				Antimicrobial activity (mm)				
			t=1	t=2	t=3	t=6	*E. faecalis*	*B.cereus*	*E.coli*	*L. monocytogenes*	*P. fluorescens*
Lactobacillus sp	A1.50.2021	+	+	-	-	-	13	16	17	20	16
Lactobacillus sp	A2.06.2022	+	+	-	-	-	13	12	22	15	10
Lactobacillus sp	A2.30.2022	+	+	-	-	-	15	12	15	20	26
Lactobacillus sp	A3.54.2021	-	+	+	+	-	18	13	13	0	15
Lactobacillus sp	A3.55.2021	+	+	+	-	-	19	18	21	0	26
Lactobacillus sp	A3.37.2022	-	+	-	-	-	14	17	25	0	22
Lactobacillus sp	A3.61.2022	-	+	+	-	-	15	18	19	0	13
Leuconosto mesenteroides	A4.8.2022	+	-	-	-	-	14	18	25	5	33
Leuconostoc mesenteroides	A4.41.2022	+	-	-	-	-	17	17	10	0	16
Lactobacillus sp	N9.3.2021	+	+	+	+	-	12	23	18	10	16
Lactobacillus sp	N9.1.2022	+	+	-	-	-	11	17	14	9	14
Lactobacillus sp	N9.29.2022	-	+	+	-	-	13	21	16	13	0
Lactobacillus sp	N9.49.2022	-	+	-	-	-	15	25	12	7	18
Leuconostoc mesenteroides	A1.64.2021	+++	+	+	-	-	5	6	7	0	0
Leuconostoc sp.	A2.12.2021	+	-	-	-	-	0	0	0	0	0

Lactobacillus plantarum	A2.24.2021	++	-	-	-	-	0	0	0	0	0
Leuconostoc sp.	A2.29.2021	-	-	-	-	-	5	9	0	4	0
Leuconostoc mesenteroides	A2.36.2021	+	+	-	-	-	7	0	11	21	0
Pediococcus sp	A3.3.2022	+	+	+	-	-	13	0	6	8	0
Leuconostoc sp.	A3.61.2022	++	-	-	-	-	0	0	0	0	0
Leuconostoc sp.	A4.5.2021	+	-	-	-	-	0	0	0	0	0
Lactobacillus sp	A4.46.2021	+++	-	-	-	-	0	0	0	0	0
Lactobacillus plantarum	A4.24.2022	+	-	-	-	-	0	0	0	0	0
Lactobacillus sp	A4.38.2022	++	-	-	-	-	0	0	0	0	0
Lactobacillus plantarum	N9.10.2022	++	-	-	-	-	0	0	0	5	0
Lactobacillus plantarum	N9.58.2022	++	-	-	-	-	0	0	0	0	0
Leuconostoc sp.	N10.59.2021	+	-	-	-	-	0	0	0	0	0
Leuconostoc sp.	N10.6.2022	++	-	-	-	-	0	0	0	0	0
Lactobacillus sp	N10.15.2022	++	-	-	-	-	0	0	0	0	0

Supplementary Table S6 - Survival screening and inhibition potential of *Enterococcus* against pathogenic and deteriorative bacteria. Positive results in bile salts and acidic pH were defined by evident bacterial multiplication. Inhibition zones against pathogenic and deteriorative bacteria were measured in millimeters.

Species ID	Isolates	Bile salts (0.5 %)	pH=2				Antimicrobial activity (mm)				
			t=1	t=2	t=3	t=6	*E. faecalis*	*B.cereus*	*E.coli*	*L. monocytogenes*	*P. fluorescens*
Enterococcus durans	A1.45 2021	+++	+	-	-	-	9	0	13	12	0
Enterococcus faecium	A2.32 2021	+++	+	+	+	-	0	0	0	6	0
Enterococcus faecalis	A2.58 2021	+++	-	-	-	-	0	0	0	6	0
Enterococcus faecalis	A3.4 2021	+++	+	+	+	-	0	11	0	0	0
Enterococcus durans	A2.1 2022	+++	+	+	+	-	5	12	0	10	0
Enterococcus faecalis	A3.47 2022	+++	+	+	+	-	0	0	0	0	0
Enterococcus durans	A4.20 2022	+++	-	-	-	-	8	0	0	7	0
Enterococcus sp.	A1.13.2021	+++	-	-	-	-	0	0	0	0	0
Enterococcus sp.	A1.8.2022	+++	+	-	-	-	14	11	9	6	10
Enterococcus faecalis	A1.55.2022	+++	+	-	-	-	28	12	10	26	12

Supplementary Table S7 - Post-hoc pairwise comparisons of aggregation percentages across incubation periods. Pairwise comparisons between incubation periods (t4, t5, and t24 hours) were performed for each isolate across all four aggregation assays: auto-aggregation, and co-aggregation with Escherichia coli, Listeria spp., and Salmonella spp. The table is organised by assay type and isolate. For each isolate, the appropriate post-hoc test was applied based on the outcome of the Shapiro-Wilk normality test: paired t-tests were used when normality was supported, and Wilcoxon signed-rank tests were applied otherwise. Raw p-values (p) and Benjamini-Hochberg false discovery rate-adjusted p-values (p.adj) are reported for each pairwise comparison (t4 vs t5, t4 vs t24, and t5 vs t24). Significance is indicated as follows: * p < 0.05, ** p < 0.01, *** p < 0.001; ns, not significant. All analyses were performed in R (version 4.4.0) using the *rstatix* package.

group 1	group 2	p-value	p.adj	p.adj.signif	Assay	Isolate
t4	t5	0.002	0.007	**	Auto-aggregation	A2.32.2021
t4	t24	0.084	0.084	ns	Auto-aggregation	A2.32.2021
t5	t24	0.016	0.023	*	Auto-aggregation	A2.32.2021
t4	t5	0.004	0.013	*	Auto-aggregation	A3.4.2021
t4	t24	0.019	0.028	*	Auto-aggregation	A3.4.2021
t5	t24	0.044	0.044	*	Auto-aggregation	A3.4.2021
t4	t5	0.032	0.032	*	Auto-aggregation	A3.47.2022
t4	t24	0.001	0.003	**	Auto-aggregation	A3.47.2022
t5	t24	0.004	0.006	**	Auto-aggregation	A3.47.2022
t4	t5	0.25	0.25	ns	Auto-aggregation	A1.8.2022
t4	t24	0.25	0.25	ns	Auto-aggregation	A1.8.2022
t5	t24	0.25	0.25	ns	Auto-aggregation	A1.8.2022
t4	t5	0.151	0.151	ns	Auto-aggregation	A1.55.2022
t4	t24	0.012	0.036	*	Auto-aggregation	A1.55.2022
t5	t24	0.024	0.036	*	Auto-aggregation	A1.55.2022
t4	t5	0.008	0.023	*	Auto-aggregation	A1.61.2022
t4	t24	0.21	0.21	ns	Auto-aggregation	A1.61.2022
t5	t24	0.016	0.024	*	Auto-aggregation	A1.61.2022
t4	t5	0.014	0.014	*	Auto-aggregation	A3.5.2021
t4	t24	0.004	0.007	**	Auto-aggregation	A3.5.2021
t5	t24	0.004	0.007	**	Auto-aggregation	A3.5.2021
t4	t5	0.02	0.02	*	Auto-aggregation	A3.54.2021
t4	t24	0.007	0.02	*	Auto-aggregation	A3.54.2021

t5	t24	0.015	0.02	*	Auto-aggregation	A3.54.2021
t4	t5	0.286	0.286	ns	Auto-aggregation	A4.35.2021
t4	t24	0.259	0.286	ns	Auto-aggregation	A4.35.2021
t5	t24	0.252	0.286	ns	Auto-aggregation	A4.35.2021
t4	t5	0.25	0.25	ns	Auto-aggregation	A4.58.2021
t4	t24	0.25	0.25	ns	Auto-aggregation	A4.58.2021
t5	t24	0.25	0.25	ns	Auto-aggregation	A4.58.2021
t4	t5	0.623	0.623	ns	Auto-aggregation	N9.3.2021
t4	t24	0.01	0.015	*	Auto-aggregation	N9.3.2021
t5	t24	0.005	0.014	*	Auto-aggregation	N9.3.2021
t4	t5	0.081	0.201	ns	Auto-aggregation	N9.1.2022
t4	t24	0.616	0.616	ns	Auto-aggregation	N9.1.2022
t5	t24	0.134	0.201	ns	Auto-aggregation	N9.1.2022
t4	t5	0.005	0.015	*	Auto-aggregation	A1.64.2021
t4	t24	0.013	0.015	*	Auto-aggregation	A1.64.2021
t5	t24	0.015	0.015	*	Auto-aggregation	A1.64.2021
t4	t5	0.102	0.102	ns	Auto-aggregation	A2.36.2021
t4	t24	0.027	0.04	*	Auto-aggregation	A2.36.2021
t5	t24	0.018	0.04	*	Auto-aggregation	A2.36.2021
t4	t5	0.075	0.075	ns	Auto-aggregation	A3.31.2021
t4	t24	0.066	0.075	ns	Auto-aggregation	A3.31.2021
t5	t24	0.059	0.075	ns	Auto-aggregation	A3.31.2021
t4	t5	0.005	0.007	**	Co-aggregation (*E. coli*)	A2.32.2021
t4	t24	0.006	0.007	**	Co-aggregation (*E. coli*)	A2.32.2021
t5	t24	0.007	0.007	**	Co-aggregation (*E. coli*)	A2.32.2021
t4	t5	0.25	0.25	ns	Co-aggregation (*E. coli*)	A3.4.2021
t4	t24	0.25	0.25	ns	Co-aggregation (*E. coli*)	A3.4.2021
t5	t24	0.25	0.25	ns	Co-aggregation (*E. coli*)	A3.4.2021
t4	t5	0.438	0.438	ns	Co-aggregation (*E. coli*)	A3.47.2022

t4	t24	0.03	0.045	*	Co-aggregation (*E. coli*)	A3.47.2022
t5	t24	0.008	0.025	*	Co-aggregation (*E. coli*)	A3.47.2022
t4	t5	0.018	0.018	*	Co-aggregation (*E. coli*)	A1.8.2022
t4	t24	0.007	0.01	**	Co-aggregation (*E. coli*)	A1.8.2022
t5	t24	0.004	0.01	**	Co-aggregation (*E. coli*)	A1.8.2022
t4	t5	0.054	0.054	ns	Co-aggregation (*E. coli*)	A1.55.2022
t4	t24	0.004	0.006	**	Co-aggregation (*E. coli*)	A1.55.2022
t5	t24	0.004	0.006	**	Co-aggregation (*E. coli*)	A1.55.2022
t4	t5	0.481	0.481	ns	Co-aggregation (*E. coli*)	A1.61.2022
t4	t24	0.047	0.07	ns	Co-aggregation (*E. coli*)	A1.61.2022
t5	t24	0.032	0.07	ns	Co-aggregation (*E. coli*)	A1.61.2022
t4	t5	0.054	0.054	ns	Co-aggregation (*E. coli*)	A3.5.2021
t4	t24	0.004	0.005	**	Co-aggregation (*E. coli*)	A3.5.2021
t5	t24	0.002	0.005	**	Co-aggregation (*E. coli*)	A3.5.2021
t4	t5	0.25	0.25	ns	Co-aggregation (*E. coli*)	A3.54.2021
t4	t24	0.25	0.25	ns	Co-aggregation (*E. coli*)	A3.54.2021
t5	t24	0.25	0.25	ns	Co-aggregation (*E. coli*)	A3.54.2021
t4	t5	0.031	0.031	*	Co-aggregation (*E. coli*)	A4.35.2021
t4	t24	0.011	0.016	*	Co-aggregation (*E. coli*)	A4.35.2021
t5	t24	0.004	0.013	*	Co-aggregation (*E. coli*)	A4.35.2021
t4	t5	0.009	0.026	*	Co-aggregation (*E. coli*)	A4.58.2021
t4	t24	0.033	0.039	*	Co-aggregation (*E. coli*)	A4.58.2021
t5	t24	0.039	0.039	*	Co-aggregation (*E. coli*)	A4.58.2021
t4	t5	0.013	0.013	*	Co-aggregation (*E. coli*)	N9.3.2021
t4	t24	0.002	0.003	**	Co-aggregation (*E. coli*)	N9.3.2021
t5	t24	0.002	0.003	**	Co-aggregation (*E. coli*)	N9.3.2021
t4	t5	0.25	0.25	ns	Co-aggregation (*E. coli*)	N9.1.2022
t4	t24	0.25	0.25	ns	Co-aggregation (*E. coli*)	N9.1.2022
t5	t24	0.25	0.25	ns	Co-aggregation (*E. coli*)	N9.1.2022
t4	t5	0.002	0.006	**	Co-aggregation (*E. coli*)	A1.64.2021

t4	t24	0.006	0.007	**	Co-aggregation (*E. coli*)	A1.64.2021
t5	t24	0.007	0.007	**	Co-aggregation (*E. coli*)	A1.64.2021
t4	t5	0.00058	0.00058	***	Co-aggregation (*E. coli*)	A2.36.2021
t4	t24	0.000213	0.00058	***	Co-aggregation (*E. coli*)	A2.36.2021
t5	t24	0.000397	0.00058	***	Co-aggregation (*E. coli*)	A2.36.2021
t4	t5	0.031	0.031	*	Co-aggregation (*E. coli*)	A3.31.2021
t4	t24	0.012	0.027	*	Co-aggregation (*E. coli*)	A3.31.2021
t5	t24	0.018	0.027	*	Co-aggregation (*E. coli*)	A3.31.2021
t4	t5	0.186	0.186	ns	Co-aggregation (*L. monocytogenes*)	A2.32.2021
t4	t24	0.002	0.005	**	Co-aggregation (*L. monocytogenes*)	A2.32.2021
t5	t24	0.003	0.005	**	Co-aggregation (*L. monocytogenes*)	A2.32.2021
t4	t5	0.025	0.03	*	Co-aggregation (*L. monocytogenes*)	A3.4.2021
t4	t24	0.004	0.012	*	Co-aggregation (*L. monocytogenes*)	A3.4.2021
t5	t24	0.03	0.03	*	Co-aggregation (*L. monocytogenes*)	A3.4.2021
t4	t5	0.319	0.319	ns	Co-aggregation (*L. monocytogenes*)	A3.47.2022
t4	t24	0.021	0.062	ns	Co-aggregation (*L. monocytogenes*)	A3.47.2022
t5	t24	0.049	0.074	ns	Co-aggregation (*L. monocytogenes*)	A3.47.2022
t4	t5	0.000538	0.000807	***	Co-aggregation (*L. monocytogenes*)	A1.8.2022
t4	t24	0.036	0.036	*	Co-aggregation (*L. monocytogenes*)	A1.8.2022
t5	t24	4.65e-05	0.00014	***	Co-aggregation (*L. monocytogenes*)	A1.8.2022
t4	t5	0.012	0.019	*	Co-aggregation (*L. monocytogenes*)	A1.55.2022
t4	t24	0.023	0.023	*	Co-aggregation (*L. monocytogenes*)	A1.55.2022
t5	t24	0.002	0.005	**	Co-aggregation (*L. monocytogenes*)	A1.55.2022
t4	t5	0.184	0.184	ns	Co-aggregation (*L. monocytogenes*)	A1.61.2022
t4	t24	0.011	0.028	*	Co-aggregation (*L. monocytogenes*)	A1.61.2022

t5	t24	0.019	0.028	*	Co-aggregation (*L. monocytogenes*)	A1.61.2022
t4	t5	0.132	0.132	ns	Co-aggregation (*L. monocytogenes*)	A3.5.2021
t4	t24	0.024	0.036	*	Co-aggregation (*L. monocytogenes*)	A3.5.2021
t5	t24	0.015	0.036	*	Co-aggregation (*L. monocytogenes*)	A3.5.2021
t4	t5	0.193	0.193	ns	Co-aggregation (*L. monocytogenes*)	A3.54.2021
t4	t24	0.017	0.025	*	Co-aggregation (*L. monocytogenes*)	A3.54.2021
t5	t24	0.005	0.016	*	Co-aggregation (*L. monocytogenes*)	A3.54.2021
t4	t5	0.902	0.902	ns	Co-aggregation (*L. monocytogenes*)	A4.35.2021
t4	t24	0.074	0.112	ns	Co-aggregation (*L. monocytogenes*)	A4.35.2021
t5	t24	0.023	0.07	ns	Co-aggregation (*L. monocytogenes*)	A4.35.2021
t4	t5	0.001	0.003	**	Co-aggregation (*L. monocytogenes*)	A4.58.2021
t4	t24	0.013	0.013	*	Co-aggregation (*L. monocytogenes*)	A4.58.2021
t5	t24	0.006	0.01	**	Co-aggregation (*L. monocytogenes*)	A4.58.2021
t4	t5	0.25	0.25	ns	Co-aggregation (*L. monocytogenes*)	N9.3.2021
t4	t24	0.25	0.25	ns	Co-aggregation (*L. monocytogenes*)	N9.3.2021
t5	t24	0.25	0.25	ns	Co-aggregation (*L. monocytogenes*)	N9.3.2021
t4	t5	0.25	0.25	ns	Co-aggregation (*L. monocytogenes*)	N9.1.2022
t4	t24	0.25	0.25	ns	Co-aggregation (*L. monocytogenes*)	N9.1.2022
t5	t24	0.25	0.25	ns	Co-aggregation (*L. monocytogenes*)	N9.1.2022
t4	t5	0.028	0.05	ns	Co-aggregation (*L. monocytogenes*)	A1.64.2021
t4	t24	0.075	0.075	ns	Co-aggregation (*L. monocytogenes*)	A1.64.2021
t5	t24	0.033	0.05	ns	Co-aggregation (*L. monocytogenes*)	A1.64.2021
t4	t5	0.018	0.036	*	Co-aggregation (*L. monocytogenes*)	A2.36.2021
t4	t24	0.024	0.036	*	Co-aggregation (*L. monocytogenes*)	A2.36.2021
t5	t24	0.036	0.036	*	Co-aggregation (*L. monocytogenes*)	A2.36.2021

t4	t5	0.014	0.021	*	Co-aggregation (*L. monocytogenes*)	A3.31.2021
t4	t24	0.115	0.115	ns	Co-aggregation (*L. monocytogenes*)	A3.31.2021
t5	t24	0.004	0.011	*	Co-aggregation (*L. monocytogenes*)	A3.31.2021
t4	t5	0.005	0.008	**	Co-aggregation (*S. enteritidis*)	A2.32.2021
t4	t24	0.006	0.008	**	Co-aggregation (*S. enteritidis*)	A2.32.2021
t5	t24	0.008	0.008	**	Co-aggregation (*S. enteritidis*)	A2.32.2021
t4	t5	0.081	0.081	ns	Co-aggregation (*S. enteritidis*)	A3.4.2021
t4	t24	0.003	0.004	**	Co-aggregation (*S. enteritidis*)	A3.4.2021
t5	t24	0.001	0.004	**	Co-aggregation (*S. enteritidis*)	A3.4.2021
t4	t5	0.076	0.079	ns	Co-aggregation (*S. enteritidis*)	A3.47.2022
t4	t24	0.079	0.079	ns	Co-aggregation (*S. enteritidis*)	A3.47.2022
t5	t24	0.078	0.079	ns	Co-aggregation (*S. enteritidis*)	A3.47.2022
t4	t5	0.000706	0.002	**	Co-aggregation (*S. enteritidis*)	A1.8.2022
t4	t24	0.162	0.162	ns	Co-aggregation (*S. enteritidis*)	A1.8.2022
t5	t24	0.014	0.021	*	Co-aggregation (*S. enteritidis*)	A1.8.2022
t4	t5	0.243	0.243	ns	Co-aggregation (*S. enteritidis*)	A1.55.2022
t4	t24	0.024	0.046	*	Co-aggregation (*S. enteritidis*)	A1.55.2022
t5	t24	0.031	0.046	*	Co-aggregation (*S. enteritidis*)	A1.55.2022
t4	t5	0.446	0.446	ns	Co-aggregation (*S. enteritidis*)	A1.61.2022
t4	t24	0.003	0.009	**	Co-aggregation (*S. enteritidis*)	A1.61.2022
t5	t24	0.012	0.018	*	Co-aggregation (*S. enteritidis*)	A1.61.2022
t4	t5	0.03	0.03	*	Co-aggregation (*S. enteritidis*)	A3.5.2021
t4	t24	0.016	0.03	*	Co-aggregation (*S. enteritidis*)	A3.5.2021
t5	t24	0.022	0.03	*	Co-aggregation (*S. enteritidis*)	A3.5.2021
t4	t5	0.25	0.25	ns	Co-aggregation (*S. enteritidis*)	A3.54.2021
t4	t24	0.25	0.25	ns	Co-aggregation (*S. enteritidis*)	A3.54.2021
t5	t24	0.25	0.25	ns	Co-aggregation (*S. enteritidis*)	A3.54.2021
t4	t5	0.722	0.722	ns	Co-aggregation (*S. enteritidis*)	A4.35.2021
t4	t24	0.051	0.077	ns	Co-aggregation (*S. enteritidis*)	A4.35.2021

t5	t24	0.02	0.06	ns	Co-aggregation (*S. enteritidis*)	A4.35.2021
t4	t5	0.034	0.051	ns	Co-aggregation (*S. enteritidis*)	A4.58.2021
t4	t24	0.014	0.04	*	Co-aggregation (*S. enteritidis*)	A4.58.2021
t5	t24	0.478	0.478	ns	Co-aggregation (*S. enteritidis*)	A4.58.2021
t4	t5	0.000308	0.000924	***	Co-aggregation (*S. enteritidis*)	N9.3.2021
t4	t24	0.002	0.003	**	Co-aggregation (*S. enteritidis*)	N9.3.2021
t5	t24	0.003	0.003	**	Co-aggregation (*S. enteritidis*)	N9.3.2021
t4	t5	0.028	0.028	*	Co-aggregation (*S. enteritidis*)	N9.1.2022
t4	t24	0.005	0.007	**	Co-aggregation (*S. enteritidis*)	N9.1.2022
t5	t24	0.003	0.007	**	Co-aggregation (*S. enteritidis*)	N9.1.2022
t4	t5	0.025	0.074	ns	Co-aggregation (*S. enteritidis*)	A1.64.2021
t4	t24	0.083	0.124	ns	Co-aggregation (*S. enteritidis*)	A1.64.2021
t5	t24	0.124	0.124	ns	Co-aggregation (*S. enteritidis*)	A1.64.2021
t4	t5	0.011	0.011	*	Co-aggregation (*S. enteritidis*)	A2.36.2021
t4	t24	0.005	0.008	**	Co-aggregation (*S. enteritidis*)	A2.36.2021
t5	t24	0.005	0.008	**	Co-aggregation (*S. enteritidis*)	A2.36.2021
t4	t5	0.003	0.009	**	Co-aggregation (*S. enteritidis*)	A3.31.2021
t4	t24	0.014	0.022	*	Co-aggregation (*S. enteritidis*)	A3.31.2021
t5	t24	0.026	0.026	*	Co-aggregation (*S. enteritidis*)	A3.31.2021
